# Persistent pulmonary pathology after COVID-19 is associated with high viral load, weak antibody response, and high levels of matrix metalloproteinase-9

**DOI:** 10.1038/s41598-021-02547-x

**Published:** 2021-12-01

**Authors:** Tøri Vigeland Lerum, Niklas Nyboe Maltzahn, Pål Aukrust, Marius Trøseid, Katerina Nezvalova Henriksen, Trine Kåsine, Anne-Ma Dyrhol-Riise, Birgitte Stiksrud, Mette Haugli, Bjørn Blomberg, Bård Reiakvam Kittang, Asgeir Johannessen, Raisa Hannula, Saad Aballi, Anders Benjamin Kildal, Ragnhild Eiken, Tuva Børresdatter Dahl, Fridtjof Lund-Johansen, Fredrik Müller, Jezabel Rivero Rodriguez, Carin Meltzer, Gunnar Einvik, Thor Ueland, Inge Christoffer Olsen, NOR-SOLIDARITY Consortium, Frank Olav Pettersen, Aleksander Rygh Holten, Kristian Tonby, Dag Henrik Reikvam, Kjerstin Røstad, Synne Jenum, Liv Hesstvedt, Eline Brenno Vaage, Anette Kolderup, Trung Tran, Jan Terje Andersen, Mona Holberg-Petersen, Simreen Kaur Johal, Åse Berg, Anders Tveita, Gernot Ernst, Lars Heggelund, Lars Thoresen, Karl Erik Müller, Dag Arne Lihaug Hoff, Roy Bjørkholt Olsen, Ane-Krisitne Finbråten, Hedda Hoel, Alexander Mathiessen, Leif Erik Vinge, Lena Bugge Nordberg, Ravinea Manotheepan, Grethe-Elisabeth Stenvik, Hans Schmidt Rasmussen, Ruth Foseide Thorkildsen, Pawel Mielnik, Lan Ai Kieu Le, Carl Magnus Ystrøm, Richard Alexander Molvik, Nina Vibeche Skei, Olav Dalgard, Bjørn Åsheim-Hansen, Anne Marie Halstensen, Jorunn Brynhildsen, Waleed Ghanima, Vegard Skogen, Krisine Greve Isdahl Mohn, Reidar Kvåle, Nina Langeland, Lise Tuset Gustad, Lars Mølgaard Saxhaug, Cathrine Fladeby, Susanne Dudman, Anne Steffensen, Andreas Barratt-Due, Trond Mogens Aaløkken, Ole Henning Skjønsberg

**Affiliations:** 1 Department of Pulmonary Medicine, Oslo University Hospital Ullevål, Nydalen, Postboks 4950, 0424 Oslo, Norway; 2 Institute of Clinical Medicine, University of Oslo, Blindern, Postboks 1171, 0318 Oslo, Norway; 3 Oslo Centre for Biostatistics and Epidemiology, Oslo University Hospital, Oslo, Norway; 4 Department of Biostatistics, Oslo Centre for Biostatistics and Epidemiology, University of Oslo, Oslo, Norway; 5 Section of Clinical Immunology and Infectious Diseases, Oslo University Hospital, Oslo, Norway; 6 Department of Haematology, Oslo University Hospital, Oslo, Norway; 7 Hospital Pharmacies of South-Eastern Norway Enterprise, Oslo, Norway; 8 Division of Critical Care and Emergencies, Oslo University Hospital, Oslo, Norway; 9 Department of Infectious Diseases, Oslo University Hospital, Ullevål, Oslo, Norway; 10 Infectious Diseases Department, Sørlandet Hospital SSK, Kristiansand, Norway; 11 Department of Medicine, Haukeland University Hospital, Bergen, Norway; 12 Department of Clinical Science, University of Bergen, Bergen, Norway; 13 Department of Medicine, Haraldsplass Deaconess Hospital, Bergen, Norway; 14 Department of Infectious Diseases, Vestfold Hospital Trust, Tønsberg, Norway; 15 Department of Infectious Diseases, Trondheim University Hospital, Trondheim, Norway; 16 Department of Infectious Diseases, Østfold Hospital Trust Kalnes, Grålum, Norway; 17 Department of Anaesthesiology and Intensive Care, University Hospital of North Norway, Tromsø, Norway; 18 Department of Infectious Diseases, Innlandet Hospital Trust, Lillehammer, Norway; 19 Division of Laboratory Medicine, Department of Immunology, Oslo University Hospital, Oslo, Norway; 20 Department of Microbiology, Oslo University Hospital, Oslo, Norway; 21 Department of Radiology and Nuclear Medicine, Oslo University Hospital Ullevål, Oslo, Norway; 22 Department of Pulmonary Medicine, Akershus University Hospital, Lørenskog, Norway; 23 Research Institute for Internal Medicine, Oslo University Hospital, Oslo, Norway; 24 Department of Research Support for Clinical Trials, Oslo University Hospital, Oslo, Norway; 25 Department of Immunology, University of Oslo, Oslo, Norway; 26 Oslo University Hospital, Nydalen, Postboks 4950, 0424 Oslo, Norway; 27 Stavanger University Hospital, Helse Stavanger HF, Postboks 8100, 4068 Stavanger, Norway; 28 Vestre Viken Hospital Trust, Vestre Viken HF, Postboks 800, 3004 Drammen, Norway; 29 Ålesund Hospital, Postboks 1600, 6026 Ålesund, Norway; 30 Sørlandet Hospital, Sørlandet Sykehus HF, Lundsiden, Postboks 416, 4604 Kristiansand, Norway; 31 Lovisenberg Diaconal Hospital, Nydalen, Postboks 4970, 0440 Oslo, Norway; 32 Diakonhjemmet Hospital, Vindern, Postboks 23, 0319 Oslo, Norway; 33 Førde Hospital, Helse Førde HF, Postboks 1000, 6807 Førde, Norway; 34 Haugesund Hospital, Helse Fonna, Postboks 2170, 5504 Haugesund, Norway; 35 Innlandet Hospital Trust, Sykehuset Innlandet, Postboks 104, 2381 Brumunddal, Norway; 36 Nord-Trøndelag Hospital Trust, Helse Nord-Trøndelag HF, Postboks 333, 7601 Levanger, Norway; 37 Akershus University Hospital, Akershus Universitetssykehus HF, Postboks 1000, Lørenskog, Norway; 38 Vestfold Hospital Trust, Vestfold HF, Postboks 2168, 3103 Tønsberg, Norway; 39 Østfold Hospital Trust, Sykehuset Østfold, Postboks 300, 1714 Grålum, Norway; 40 University Hospital of North-Norway, Sykehusvegen 38, 9091 Tromsø, Norway; 41 Haukeland University Hospital, Haukeland Universitetssjukehus, Postboks 1400, 5021 Bergen, Norway

**Keywords:** Predictive markers, Respiratory distress syndrome, Viral infection, Antimicrobial responses

## Abstract

The association between pulmonary sequelae and markers of disease severity, as well as pro-fibrotic mediators, were studied in 108 patients 3 months after hospital admission for COVID-19. The COPD assessment test (CAT-score), spirometry, diffusion capacity of the lungs (DL_CO_), and chest-CT were performed at 23 Norwegian hospitals included in the NOR-SOLIDARITY trial, an open-labelled, randomised clinical trial, investigating the efficacy of remdesivir and hydroxychloroquine (HCQ). Thirty-eight percent had a CAT-score ≥ 10. DL_CO_ was below the lower limit of normal in 29.6%. Ground-glass opacities were present in 39.8% on chest-CT, parenchymal bands were found in 41.7%. At admission, low pO_2_/F_i_O_2_ ratio, ICU treatment, high viral load, and low antibody levels, were predictors of a poorer pulmonary outcome after 3 months. High levels of matrix metalloproteinase (MMP)-9 during hospitalisation and at 3 months were associated with persistent CT-findings. Except for a negative effect of remdesivir on CAT-score, we found no effect of remdesivir or HCQ on long-term pulmonary outcomes. Three months after hospital admission for COVID-19, a high prevalence of respiratory symptoms, reduced DL_CO_, and persistent CT-findings was observed. Low pO_2_/F_i_O_2_ ratio, ICU-admission, high viral load, low antibody levels, and high levels of MMP-9 were associated with a worse pulmonary outcome.

## Introduction

The abrupt spread of SARS-CoV-2, leading to the COVID-19 pandemic, has had a devastating impact on the entire world’s population^1^. With the lungs as the primary target, knowledge about the prevalence and causes of long-term pulmonary sequelae in COVID-19 survivors is paramount.

We recently reported that 50% of COVID-19 survivors struggle with dyspnoea 3 months following hospital admission, with 25% presenting with reduced diffusion capacity of the lungs for carbon monoxide (DL_CO_), and 20% having signs of early pulmonary fibrosis on chest computed tomography (CT)^2^. Similar results have been reported by others^3^. With various drugs emerging as possible therapeutic candidates against SARS-CoV-2, long-term pulmonary function following treatment and potential targets for preventing pulmonary sequelae warrant elucidation^4^.

The NOR-SOLIDARITY trial is an independent add-on study to the WHO SOLIDARITY trial, evaluating the effects of remdesivir and hydroxychloroquine (HCQ) compared to standard of care (SoC) in hospitalised COVID-19 patients, assessed by in-hospital mortality, the need for mechanical ventilation and the length of hospital stays^5^. To explore end-organ deterioration following the infection, the NOR-SOLIDARITY trial included biobanking during hospital stay and at a 3-month follow-up visit^6^.

Studies of patients hospitalised for SARS, caused by another beta coronavirus (SARS-CoV) with high similarity to SARS-CoV-2, have highlighted the role of an overwhelming host response in mediating lung injury and subsequent development of pulmonary fibrosis^7^. In other forms of pulmonary fibrosis, several mediators are under investigation as markers and potential mediators of disease severity and prognosis, such as various matrix metalloproteinases (MMP), surfactant protein D (SP-D), and vascular endothelial growth factor (VEGF) A, as a key regulator of angiogenesis^8–10^. Some of these mediators have also been reported to be elevated in the acute phase of COVID-19^11,12^. However, the association between these regulators of extracellular matrix remodelling and the development of persistent lung parenchyma pathology and reduced pulmonary function following COVID-19, has not been fully elucidated.

We aimed to investigate pulmonary function through COPD Assessment Test Score (CAT-score), spirometry, DL_CO_, and chest CT 3 months after hospital admission for COVID-19, and whether there was a difference in these variables between participants treated with remdesivir, HCQ, or local SoC alone. As explorative outcomes, we evaluated associations between clinical characteristics during hospital admission, including viral load in oropharynx and the presence of antibodies against SARS-CoV-2, with the pulmonary outcomes. In addition, we examined if plasma levels of factors suspected to be involved in development of pulmonary fibrosis (i.e., MMP-9, SP-D, and VEGF-A), obtained during hospital admission and after 3 months, were related to patient-reported outcome measures, as well as pulmonary function and persistent findings on chest CT.

## Materials and methods

### Design

The NOR-SOLIDARITY trial is an adaptive, multicentred, randomised, open-labelled clinical trial based on the WHO trial SOLIDARITY, where the target was to evaluate the efficacy and safety of remdesivir and HCQ versus their respective SoC group in hospital-admitted COVID-19 patients. A 3-month follow-up visit was organized to evaluate lung function by spirometry, DL_CO_, and chest CT. Peripheral blood was collected both during hospital stay and at the 3-month follow up. Biobanking material from oropharynx was collected during hospital admission. Recent work by the SOLIDARITY and NOR-SOLIDARITY consortia describes in detail the design of the trial, the recruitment of participants, the randomisation process, interventions, and secondary outcomes^5,6^. The study was approved by the Committee for Medical Research Ethics Region South East Norway (REK 118684) and registered on ClinicalTrials.gov on the 25.03.2020 (NCT04321616). Monitors from the Clinical Trial Unit at Oslo University Hospital monitored the conduct of the trial. Good clinical practice was followed throughout the study. All participants gave informed consent prior to inclusion, either themselves or by a legally authorised representative.

### Outcomes

The outcomes of this sub-study were CAT-score, spirometry, DL_CO_, and chest CT-findings 3 months following hospital admission for COVID-19, and the effect of remdesivir and HCQ versus their respective SoC on these variables. Explorative outcomes were to correlate clinical characteristics during hospital admission with the pulmonary outcomes. These included pO_2_/F_i_O_2_ (P/F)-ratio, respiratory rate (RR), admission to an intensive care unit (ICU), the receipt of mechanical ventilation, as well as plasma levels of C-reactive protein (CRP), lactate dehydrogenase (LDH), D-dimer, ferritin, viral load in oropharynx, and antibodies against the receptor binding domain (RBD) of SARS-CoV-2^13^. Secondary explorative outcomes were to correlate the levels of MMP-9, SP-D, and VEGF-A during hospital admission and at 3 months following discharge for COVID-19, with the pulmonary outcomes.

### Participants

Twenty-three Norwegian hospitals participated in the NOR-SOLIDARITY trial. Adult patients (≥ 18 years) admitted to hospital with a PCR-confirmed SARS-CoV-2 infection from March 28th until October 5th 2020 were eligible for participation. The exclusion criteria are described in detail in a recently published report^6^. For the purpose of this report, participants having completed the 3-month follow-up visit by the 15th of October 2020 were included (n = 108). Participants were randomised to receive remdesivir, HCQ or SoC. During the trial period, the availability of the drugs varied, implying that some participants served as SoC control subjects for one drug, while others served as SoC control subjects for both remdesivir and HCQ. This might give the impression that there are more than 108 participants included. An overview of missing parameters (i.e., CT scans, blood analyses, and pulmonary function tests) are presented in Supplementary Table S1.

### Intervention

In the NOR-SOLIDARITY trial, the participants were allocated to one of three treatment arms; SoC, remdesivir (200 mg day 1, then 100 mg daily up to nine days), or HCQ (800 mg twice daily day 1, then 400 mg twice daily up to nine days). All trial regiments were discontinued at discharge. Unintended effects are described in a previous report^6^.

### Patient-reported outcome measures

CAT-score is an eight-item questionnaire designed to assess and quantify the impact of COPD symptoms on health status on a scale from 0 (low) to 40 (very high)^14^. The eight items cover respiratory symptoms (cough, mucus, tightness in chest) and impaired quality of life (limitations in daily activities, sleep impairment, loss of energy). Scores ≥ 10, indicating a moderate to very high symptom impact on health status, were used as a cut-off^15^. Participants responded to the questionnaire at the 3-month follow-up visit.

### Pulmonary function tests

Pulmonary function tests consisted of spirometry (Jaeger, Höechberg, Germany and Carefusion, Yorba Linda, CA, USA) and DL_CO_, and were performed according to the European Respiratory Society’s and the American Thoracic Society’s guidelines, thoroughly described in detail in a previously published report^2,16–19^.

### Acquisition and review of chest CT images

Low-dose, thin-section chest CT images were obtained in supine and prone position during breath-holding in deep inspiration. Supplementary expiratory scans were obtained in 18 (17.6%) participants to differentiate between air trapping and hypoperfusion in areas with decreased attenuation on inspiratory scans. The CT protocol and method of interpretation according to the Fleischner Society, are described in detail in a previously published report^2,20,21^. For the purpose of this report, we assessed the prevalence of any ground-glass opacities (GGO), GGO ≥ 10% in at least one of the four lung zones, or any mosaic pattern (classified as potentially reversible changes and grouped together), any consolidations, reticular pattern, parenchymal bands, interlobular septal thickening, or any bronchiectasis (classified as irreversible changes and grouped together).

### Routine laboratory analyses and measurements of SARS-COV-2 viral load and antibodies

Routine blood samples (CRP, LDH, ferritin, D-dimer, white blood cell counts, the number of neutrophils, lymphocytes, and platelets, and haemoglobin levels) were analysed by the routine laboratory at each participating centre. Viral load in oropharynx was analysed by RT-PCR, and SARS-CoV-2 receptor binding domain (RBD) IgG antibodies were detected by multiplexed bead-based flow cytometric assay, referred to as microsphere affinity proteomics as previously described^6^.

### Measurement of MMP-9, tissue inhibitor of metalloproteinase (TIMP) 2, SP-D, AND VEGF-A

Per protocol, biobanking was performed during hospital admission and at the 3-month follow up visit. Plasma was collected in sterile EDTA containing tubes, put on melting ice and centrifuged at 2000×*g* for 20 min to obtain platelet-poor plasma, before storage at − 80 °C. The samples were thawed only once. Plasma levels of MMP-9, TIMP-2, SP-D, and VEGF-A were measured in duplicate by enzyme immunoassays (EIA) using commercially available reagents (Cat#DY911, DY971, DY1920, DY293B; RnD systems, Stillwater, MN, USA) in a 384-well format. Absorption was read at 450 nm with wavelength correction set to 540 nm using an EIA plate reader (BioTek). Intra- and inter-assay coefficients of variation were < 10% for all EIAs. Levels of MMP-9, TIMP-2, SP-D, and VEGF-A in the plasma of 24 healthy blood donors (15 (62.5%) males, mean age 44 years, one (4.2%) former smoker, one (4.2%) taking statins), were also measured to establish a point of reference.

### Statistics

The primary outcome of the study design has already been published, and analyses in this report are to be considered as secondary and explorative analyses^5,6^. Demographic data were described by median (25th and 75th percentile (IQR)) and percentages. CAT-score, pulmonary function tests, and CT-findings at the 3-months follow-up are presented as median values and IQR. We assessed the difference in pulmonary function, defined by FEV_1_ and DL_CO_ in percent of predicted (DL_CO_%) as continuous variables, CAT-score, and CT-findings between the intervention groups and their respective SoC-group 3 months following treatment for COVID-19 by 95% confidence intervals from the two-sample t-distribution, or p-value from the 2 × 2 Chi-squared distribution. To evaluate participants’ symptom load to pulmonary function, we associated CAT-score ≥ 10 with DL_CO_%, and the presence of any reversible, and any irreversible CT-findings using logistic regression.

Associations between clinical characteristics during hospital stay and pulmonary outcomes after 3 months, were performed by logistic and linear regression, and adjusted by age, sex, treatment, and history of smoking. As an explorative outcome we wanted to study whether these clinical characteristics could predict pulmonary dysfunction after 3 months, defined by CAT-score ≥ 10, DL_CO_%, and the presence of either GGO in more than ≥ 10% of at least one of four lung zones as a measure of possible reversible changes, and irreversible changes on chest CT.

Further explorative outcomes were associations between the maximum plasma levels of MMP-9, TIMP-2, SP-D, and VEGF-A during hospital stay and at the 3-month follow-up visit, with DL_CO_%, and the predefined changes on chest CT. Comparisons of pro-fibrotic mediators between the healthy control group, the maximum levels during admission and at the 3-months follow-up, were performed with Mann–Whitney U test and Wilcoxon paired test. Statistical analyses were performed using the statistical software R (version 4.0.2, 2020.06.22, R Core Team (2020). R: A language and environment for statistical computing. R Foundation for Statistical Computing, Vienna, Austria. URL https://www.R-project.org/) and SPSS (IBM Corp. released 2019. IBM SPSS Statistics for Windows, Version 26.0. Armonk, NY: IBM Corp). Figures were produced using Prism 9 (GraphPad Software, LCC, version 9.1.0., San Diego, California, USA).

## Results

### Participant flow

**Figure 1:**
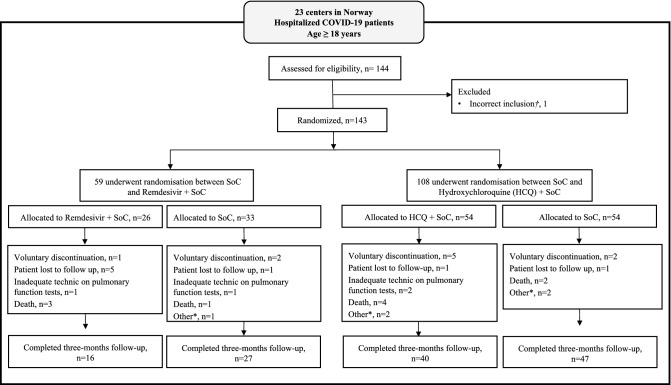
Flow chart of the participant flow. *Other: Emigration, progression of cancer diseases, general health deterioration, prolonged corrected QT-time during treatment with HCQ; ^†^Excluded from the full analysis set. *HCQ* hydroxychloroquine.

Hospitalised COVID-19 patients were included from 23 Norwegian hospitals from the 28th of March 2020. For the purpose of this paper, the data collection from the 3-month follow up visit was ended on the 15th of October, 2020. As demonstrated in Fig. 1, 108 participants completed the 3-month follow-up visit, with a median time of 93.5 (IQR 88, 106) days from hospital admission. Missing parameters are presented in Supplementary Table S1.

### Demographics

Demographic and clinical characteristics from the hospital admission for all participants and treatment groups with their respective SoC-group are presented in Table 1. Overall, the median age was 58 years (range 27.4, 89.8), 41 (38%) were female, few were current smokers (1.9%), and the median body mass index (BMI) was 27.6 (IQR 25.3, 30.7). Of comorbidities, hypertension (31.5%), diabetes mellitus (15.7%), and obesity (25.9%) were the most common. Overall, 40 (37.0%) presented with respiratory failure at inclusion, defined as P/F-ratio ≤ 40 kPa. The median duration of symptoms prior to hospital admission was eight days (IQR 5, 10).

**Table 1:** Characteristics of population at baseline.

	All participants	Remdesivir versus its control		HCQ versus its control	
	(N = 108)	Remdesivir + SoC (n = 16)	SoC (n = 27)	HCQ + SoC (n = 40)	SoC (n = 47)
**Demographics**					
Age, years	58.0 (50.0, 65.3)	58.0 (52.3, 62.0)	61.0 (47.5, 68.5)	55.0 (50.8, 74.0)	61.0 (47.5, 69.5)
Female, n (%)	41 (38.0)	6 (37.5)	6 (22.2)	17 (42.5)	18 (38.3)
Body Mass Index (kg/m^2^)	27.6 (25.3, 30.7)	27.3 (22.9, 32.6)	27.2 (25.3, 28.3)	27.8 (26.7, 31.8)	27.2 (25.0, 28.9)
Smoking history					
Current, n (%)	2 (1.9)	1 (6.2)	0 (0)	1 (2.5)	0 (0)
Previous, n (%)	40 (37.0)	6 (37.5)	12 (44.4)	14 (35.0)	18 (38.3)
Never, n (%)	62 (57.4)	7 (43.7)	14 (51.8)	25 (62.5)	27 (57.4)
**Comorbidities**					
Chronic pulmonary disease, n (%)	5 (4.6)	2 (12.5)	0 (0)	2 (5.0)	1 (2.1)
Hypertension, n (%)	34 (31.5)	5 (31.2)	9 (33.3)	11 (27.5)	17 (36.2)
Diabetes mellitus, n (%)	17 (15.7)	4 (25.0)	1 (3.7)	6 (15.0)	7 (14.9)
Obesity (BMI > 30 kg/m^2^), n (%)	28 (25.9)	6 (37.5)	2 (7.4)	14 (35.0)	8 (17.0)
**Baseline characteristics**					
Duration of symptoms prior to baseline (days)	8 (5.0, 10.0)	8 (6.7, 9.5)	8.0 (4.5, 9.0)	8.0 (5.0, 10.0)	8.0 (4.5, 9.5)
P/F-ratio at admission (kPa)	42.4 (32.5, 49.6)	42.8 (30.4, 52.2)	45.2 (36.9, 50.8)	45.2 (31.6, 48.1)	43.2 (35.8, 51.9)
P/F-ratio < 40 kPa, n (%)	40 (37.0)	6 (37.5)	10 (37.0)	18 (45.0)	14 (29.8)
Respiration rate per minute	21 (18, 24)	21 (17.5, 24.0)	20 (18.5, 24.0)	20.5 (16.0, 24.0)	21 (18.5, 24.0)
Temperature (°C)	37.3 (36.8, 38.0)	37.1 (36.7, 37.6)	37.3 (36.7, 37.8)	37.5 (36.9, 38.2)	37.2 (36.9, 37.9)
Admission to ward, n (%)	100 (92.6)	14 (87.5)	27 (100.0)	35 (87.5)	46 (97.9)
Admission to ICU, n (%)	8 (7.4)	2 (12.5)	0 (0)	5 (12.5)	1 (2.1)
**Biochemical presentation**					
Hemoglobin (g/dL)	13.4 (12.2, 14.1)	13.4 (12.2, 14.1)	13.6 (12.5, 14.2)	13.3 (12.2, 14.7)	13.3 (12.4, 14.1)
White blood cell count (× 10^9^ per L)	6.1 (4.6, 8.9)	5.5 (30, 6.6)	6.2 (4.8, 8.4)	6.7 (4.5, 9.4)	6.1 (4.9, 8.3)
Lymphocytes (× 10^9^ per L)	1.1 (0.8, 1.4)	1.0 (0.7, 1.53)	1.0 (0.7, 1.9)	1.0 (0.8, 1.3)	1.1 (0.9, 1.4)
Platelet count (× 10^9^ per L)	205 (163, 292)	194 (171, 294)	216 (162, 272)	198 (155, 292)	206 (165, 273)
Creatinine (µmol/L)	73.0 (57.0, 86.5)	62.0 (53.0, 77.0)	78.0 (59.0, 89.0)	73.0 (58.0, 90.0)	73.0 (56.5, 83.5)
Ferritin (µg/L)	627 (322, 1190)	637 (386, 1420)	627 (320, 1060)	710 (276, 1390)	532 (322, 990)
Lactate dehydrogenase (U/L)	275 (209, 357)	280 (223, 321)	214 (188, 357)	276 (228, 367)	255 (192, 353)
CRP (mg/L)	70.0 (34.8, 124.0)	79.5 (24.8, 141.0)	84.0 (31.5, 126)	71.5 (46.8, 122.0)	66.0 (30.5, 119.0)
**Viral load (oropharynx)**					
Viral load (log_10_ counts/1000 cells)	1.73 (0.00, 0.48)	1.85 (0.00, 4.51)	1.36 (0.00, 4.83)	1.88 (0.00, 4.27)	1.53 (0.00, 4.83)
**Anti-SARS-CoV-2 antibodies**					
Seroconverted (RBD ≥ 5), n (%)	42 (24.1)	5 (31.2)	11 (40.7)	13 (32.5)	20 (42.6)
RBD (AU/mL)	5.87 (2.26, 35.1)	2.65 (1.79, 7.33)	5.87 (2.41, 37.2)	3.07 (2.38, 28.6)	6.31 (2.21, 37.3)

Median values (25th–75th percentile) unless otherwise specified.

*HCQ* hydroxychloroquine, *SoC* standard of care, *ICU* intensive care unit, *P/F ratio* arterial oxygen pressure (pO_2_) divided by fraction of inspired oxygen (f_i_O_2_), *CRP* C-reactive protein, *RBD* receptor binding domain.

### CAT-score, pulmonary function tests, and chest ct-findings 3 months following treatment for COVID-19

**Figure 2:**
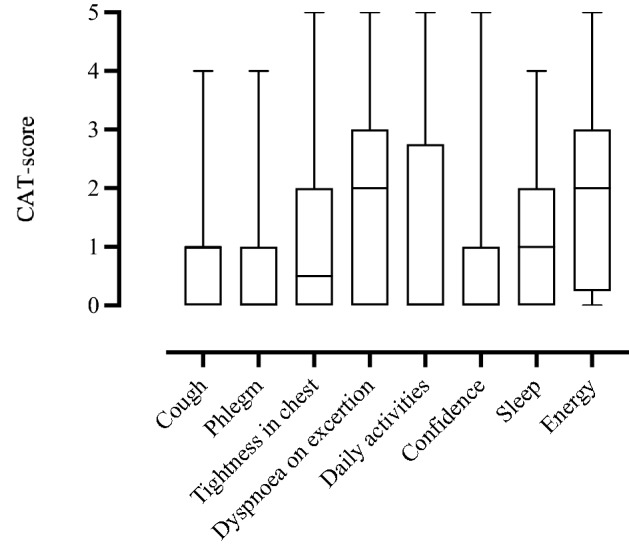
The CAT-score results for the overall population 3 months following hospital admission for COVID-19. CAT-score is an eight-item questionnaire designed to assess and quantify the impact of COPD symptoms on health status. The items comprise symptoms of chough, phlegm, feeling of chest tightness, breathlessness, limitations in activities, confidence, sleep problems, and energy. Each item ranging from 0 = no symptoms, to 5 = high symptom burden. *CAT-score* COPD Assessment Test score, *COPD* chronic obstructive pulmonary disease.

Table 2 presents CAT-score, pulmonary function tests, and all registered CT-findings 3 months following hospital admission for the total COVID-19 population, as well as the distribution between the intervention groups and their respective SoC groups. As demonstrated in Table 3, there was a higher prevalence of self-reported symptoms in the remdesivir group than in the corresponding SoC group. Otherwise, there were no significant differences between the intervention groups and their respective SoC groups with regard to pulmonary function tests or CT-findings 3 months following inclusion. Forty-one (38.0%) participants had a CAT-score ≥ 10, indicating a moderate to high symptom burden 3 months following hospitalisation (Table 2), with dyspnoea on exertion and loss of energy being the most prominent symptoms (Fig. 2).

**Table 2:** Presentation of lung function and CT findings 3 months after treatment for COVID-19.

	All patients	Remdesivir versus its control		HCQ versus its control	
	(n = 108)	Remdesivir + SoC (n = 16)	SoC (n = 27)	HCQ + SoC (n = 40)	SoC (n = 47)
**CAT-score**					
Score	8.0 (3.8, 12.3)	13.0 (8.5, 21.5)	7.0 (2.5, 10.5)	6.5 (2.25, 13.8)	8.0 (4.25, 10.0)
Score ≥ 10, n (%)	41 (41.3%)	9 (56.2)	7 (25.9)	13 (32.5)	11 (23.4)
**Spirometry**					
FVC, L	3.68 (2.89, 4.48)	3.73 (3.07, 4.61)	4.25 (3.29, 4.57)	3.62 (2.84, 4.45)	3.60 (2.85, 4.37)
FVC, % of predicted	91.4 (81.3, 102.0)	89.4 (77.2, 97.0)	94.5 (83.9, 105.0)	90.3 (80.9, 98.3)	92.8 (83.0, 106.0)
FVC < LLN, n (%)	13.0 (12.0)	2 (12.5)	3 (11.1)	6 (15)	5 (10.6)
FEV_1_, L	2.92 (2.32, 3.51)	2.97 (2.39, 3.51)	3.10 (2.55, 3.57)	2.87 (2.37, 3.53)	2.84 (2.22, 3.45)
FEV_1_, % of predicted n (%)	90.3 (81.5, 100.0)	87.3 (79.9, 91.9)	98.0 (83.8, 102.0)	90.4 (82.0, 99.9)	92.5 (82.1, 102.0)
FEV_1_ < LLN, n (%)	15 (13.9)	2 (12.5)	3 (11.1)	6 (15.0)	6 (12.8)
FEV_1_/FVC, %	79.0 (74.0, 82.0)	79.0 (74.5, 81.0)	78.0 (73.0, 81.0)	80.0 (76.0, 82.8)	78.0 (73.0, 83.0)
**Diffusion capacity**					
DL_CO_, mmol/kPa/min	6.82 (5.51, 8.42)	6.88 (5.45, 7.88)	7.56 (5.86, 8.71)	6.97 (5.82, 8.50)	6.48 (5.42, 8.19)
DL_CO_, % of predicted	84.7 (72.2, 94.1)	77.6 (67.3, 86.9)	83.4 (72.7, 95.9)	88.4 (71.4, 95.1)	85.1 (72.7, 93.1)
DL_CO_ < LLN, n (%)	32 (29.6)	6 (37.5)	7 (25.9)	12 (30.0)	14 (29.8)
KCO, mmol/kPa/min/L	1.39 (1.23, 1.55)	1.36 (1.15, 1.52)	1.31 (1.15, 1.51)	1.43 (1.30, 1.54)	1.31 (1.10, 1.59)
KCO, % of predicted	97.4 (84.1, 107.0)	96.1 (78.1, 99.8)	92.8 (83.2, 104.0)	102.0 (92.0, 108.0)	92.8 (79.4, 107.0)
KCO < LLN, n (%)	14 (13.0)	3 (18.8)	4 (14.8)	3 (7.5)	8 (17.0)
**Computed tomography**					
Possible reversible changes					
GGO, any, n (%)	43 (39.8)	6 (37.5)	13 (48.1)	17 (42.5)	17 (36.2)
GGO > 10%, n (%)	19 (17.6)	2 (12.5)	4 (14.8)	11 (27.5)	6 (12.8)
Mosaic pattern, any	26 (24.1)	7 (25.9)	1 (6.2)	11 (27.6)	12 (25.5)
Possible irreversible changes					
Parenchymal bands, any, n (%)	45 (41.7)	6 (37.5)	14 (51.9)	16 (40.0)	20 (42.6)
Reticular pattern, any, n (%)	10 (9.3)	1 (6.2)	0 (0)	5 (12.5)	4 (8.5)
Interlobular septal thickening, n (%)	16 (14.8)	2 (12.5)	3 (11.1)	9 (22.5)	4 (8.5)
Consolidations, any, n (%)	5 (4.6)	2 (12.5)	0 (0)	2 (5.0)	1 (2.1)
Bronchiectasis, any, n (%)	5 (4.6)	1 (6.2)	0 (0)	3 (7.5)	1 (2.1)

Median (25th–75th percentile) unless otherwise specified.

*HCQ* hydroxychloroquine, *SoC* standard of care, *FVC* forced vital capacity, *LLN* lower limit of normal, *FEV*_*1*_ forced expiratory volume in the 1st second, *DL*_*CO*_ diffusion capacity of the lungs for carbon monoxide, *KCO* transfer coefficient of the lungs for carbon monoxide, *CAT-score* the COPD assessment test, *GGO* ground-glass opacities.

GGO ≥ 10% in ≥ one lung zone.

**Table 3:** Effect of medication on patient reported outcomes, lung function and CT-findings 3 months after intervention.

Remdesivir vs its SoC	Remdesivir + SoC (n = 16)	SoC (n = 27)	Coefficient estimate (95% confidence interval)
**Lung function**			
FEV_1_, (%)	87.3 (79.9, 91.9)	98.0 (83.8, 102.0)	1.54 (− 0.02, 0.15)
DL_CO_, (%)	77.6 (67.3, 86.9)	83.4 (72.7, 95.9)	1.44 (− 3.03, 17.80)
**CT findings**			
Reversible (%)	3 (18.8)	4 (14.8)	Chi squared 0.40. df = 2, p = 0.82
Irreversible (%)	14 (51.9)	14 (51.9)	
**Patient reported outcomes**			
CAT-score	13.0 (4.0, 28.0)	7.0 (0, 22.0)	− 3.36 (− 12.63, − 3.00)**

HCQ vs its SoC	HCQ + SoC (n = 40)	SoC (n = 47)	Coefficient estimate (95% confidence interval)
**Lung function**			
FEV_1_, (%)	90.4 (82.0, 99.9)	92.5 (82.1, 102.0)	0.66 (− 0.04, 0.08)
DL_CO_, (%)	88.4 (71.4, 95.1)	85.1 (72.7, 93.1)	0.06 (− 7.83, 8.34)
**CT findings**			
Reversible (%)	8 (20.0)	7 (14.9)	Chi squared 0.90, df 2, p = 0.64
Irreversible (%)	19 (47.5)	22 (46.8)	
**Patient reported outcomes**			
CAT-score	6.5 (2.25, 13.8)	8.0 (4.25, 10.0)	− 0.66 (− 3.74, 1.89)

Median values (25th, 75th percentile) unless otherwise specified. Comparison done by Welch t-test for continuous variables and Pearson’s Chi squared test for categorical variables.

Reversible CT findings defined as presence of any ground glass opacities and any mosaic pattern. Irreversible CT findings defined as presence of any parenchymal bands, consolidations, reticular pattern, interlobular septal thickening, or bronchiectasis.

*SoC* standard of care, *FEV*_*1*_ forced expiratory volume in 1 s, in percent of predicted, *DL*_*CO*_ diffusion capacity of the lungs for carbon monoxide, in percent of predicted, *CT* computed tomography, *CAT* the COPD assessment test, *HCQ* hydroxychloroquine, *df* degrees of freedom.

**p < 0.01.

For the pulmonary function tests, the majority of participants had values within the limits of normal. For FVC and FEV_1_, 13 (12%) and 15 (13.9%) of the overall population had values below LLN, respectively. Thirty-two (29.6%) had DL_CO_ below LLN.

The most dominant finding on chest CT was parenchymal bands, a potential sign of pulmonary fibrosis, present in 45 (41.7%) participants. GGO, suggesting sustained inflammation, were also a prevalent finding in the total study population; 43 participants (39.8%). GGO present in ≥ 10% in one of the four lung zones, were found in 19 (17.6%) participants in the overall population.

### CAT-score associated with DL_CO_% and chest CT-findings

No association between pulmonary-related symptomatology at 3 months, determined by CAT-score, and pulmonary function tests and pathology on chest CT at the same point, was observed (Supplementary Table S2).

### Association between clinical characteristics during hospital admission and pulmonary outcomes

Table 4 presents explorative associations between demographic, clinical and biochemical characteristics at baseline and the pulmonary outcomes after 3 months, adjusted for age, sex, treatment, and a history of smoking.

**Table 4:** Association of respiratory symptoms, DL_CO_, ground glass opacities, and irreversible CT-findings, with clinical and demographic characteristics during hospital admission.

	Respiratory symptoms (CAT-score ≥ 10)^#^	DL_CO_ (% of predicted)^#^	GGO (> 10% in ≥ 1 lung zone)^#^	Irreversible CT findings (yes vs no)^#^
	Coefficient estimate (95% CI)	Coefficient estimate (95% CI)	Coefficient estimate (95% CI)	Coefficient estimate (95% CI)
Female	− 0.30 (− 1.23, 0.59)	− 1.11 (− 8.05, 5.83)	− 1.56 (− 3.21, − 0.24)*	− 0.95 (− 1.90, − 0.04)*
Age	− 0.02 (− 0.06, 0.01)	− 0.40 (− 0.65, − 0.16)**	0.07 (0.02, 0.13)**	0.07 (0.03, 0.11)***
History of smoking	1.14 (0.26, 2.06)*	3.99 (− 2.79, 10.77)	0.15 (− 1.00, 1.27)	0.23 (− 0.66, 1.12)
P/F ratio	< − 0.01 (− 0.03, 0.03)	0.31 (0.05, 0.57)*	− 0.03 (− 0.08, 0.01)	− 0.01 (− 0.05, 0.02)
RR	0.75 (− 0.18, 1.70)	0.59 (− 6.57, 7.75)	0.66 (− 0.51, 1.85)	− 0.24 (− 1.19, 0.69)
Mechanical ventilation	− 0.56 (− 2.20, 0.83)	− 10.21 (− 20.76, 0.34)	0.66 (− 0.93, 2.14)	0.49 (− 0.88, 2.01)
ICU admission	1.50 (− 0.24, 3.58)	− 14.66 (− 27.65, − 1.68)*	1.46 (− 0.20, 3.17)	1.11 (− 0.55, 3.15)
CRP	< − 0.01 (− 0.01, < 0.01)	0.01 (− 0.03, 0.06)	< 0.01 (< − 0.01, 0.01)	< − 0.01 (− 0.01, < 0.01)
D-dimer	0.09 (− 0.14, 0.53)	0.05 (− 1.57, 1.66)	0.31 (− 0.01, 1.00)	0.05 (− 0.16, 0.37)
LDH	< 0.01 (< − 0.01, < 0.01)	− 0.01 (− 0.04, 0.01)	< 0.01 (< − 0.01, 0.01)	< 0.01 (< − 0.01, 0.01)
Ferritin	< 0.01 (< − 0.01, < 0.01)	< − 0.01 (< − 0.01, < 0.01)	< − 0.01 (< − 0.01, < 0.01)	< 0.01 (< − 0.01, < 0.01)
Viral load	0.19 (− 0.20, 0.59)	− 3.61 (− 6.15, − 1.08)**	− 0.40 (− 1.06, 0.15)	− 0.13 (− 0.54, 0.26)
SARS-CoV-2 antibodies (RBD ≥ 5)	− 0.03 (− 1.06, 1.00)	13.54 (6.17, 20.90)***	0.32 (− 1.20, 1.88)	0.10 (− 0.92, 1.10)

Main effect linear logistic regression analyses, adjusted for treatment, sex, age, and history of smoking.

Biochemical markers are baseline values.

Irreversible CT findings defined as any finding of parenchymal bands, consolidations, reticular pattern, or interlobular septal thickening.

*DL*_*CO*_ diffusion capacity of the lungs for carbon monoxide, *CAT score* the COPD Assessment Test score, *RR* respiratory rate, *p/f ratio* pO2/FiO2 ratio, *ICU* intensive care unit, *CRP* C-reactive protein, *LDH* lactate dehydrogenase, *RBD* receptor binding domain, *GGO* ground glass opacities, *CT* computed tomography.

*p < 0.05, **p < 0.01, ***p < 0.001.

^#^Dependent variable.

CAT-score ≥ 10 demonstrated a significant positive correlation with a history of smoking. Three-months’ values of DL_CO_%, the most frequently affected pulmonary function test variable, were positively correlated with P/F-ratio, negatively correlated with admission to ICU, negatively correlated with viral load at baseline, and positively correlated with the presence of IgG antibodies against SARS-CoV-2 RBD at baseline. Both the presence of GGO in ≥ 10% in at least one of the lung zones and the presence of irreversible CT-findings at 3 months were negatively correlated with female sex and positively correlated with age. Notably, we found no association between markers of inflammation during hospital admission (i.e., CRP, LDH, D dimer, and ferritin) and pulmonary outcomes at 3 months (Table 4).

### Association of pro-fibrotic mediators with DL_CO_% and chest CT-findings

**Figure 3:**
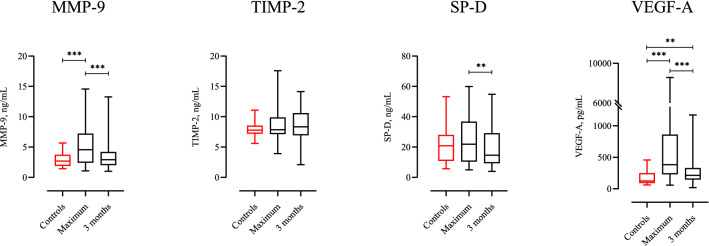
Group comparison of pro-fibrotic markers between 24 healthy control subjects, and all participants’ maximum plasma levels during hospital stay and at the 3-months follow-up evaluation, performed with Mann–Whitney U test and Wilcoxon paired test. Controls, 24 healthy control subjects. Maximum, maximum plasma levels during the hospital stay. 3 months, plasma levels at the 3-month follow-up. *MMP-9* matrix metalloproteinase 9, *TIMP-2* tissue inhibitor of metalloproteinases 2, *SP-D* surfactant protein D, *VEGF-A* vascular endothelial growth factor. Median MMP-9 level healthy control subjects: 2.70 ng/mL, median maximum level MMP-9: 3.83 ng/mL, median MMP-9 level at 3 months: 2.93 ng/mL. Median TIMP-2 level healthy control subjects: 7.78 ng/mL, median maximum level TIMP-2: 7.85 ng/mL, median TIMP-2 level at 3 months: 8.76 ng/mL. Median SP-D level healthy control subjects: 20.9 ng/mL, median maximum level SP-D 16.4, median SP-D level at 3 months: 14.1 ng/mL. Median VEGF-A level healthy control subjects: 125 pg/mL, median maximum VEGF-A level: 277 pg/mL, median VEGF-A level at 3 months: 216 pg/mL. *p < 0.05, **p < 0.01, ***p < 0.001.

The maximum levels of both MMP-9 and VEGF-A during the hospital stay were significantly higher among participants, than in the healthy control group (Fig. 3). All mediators showed a significant decrease from the acute phase to the 3-month follow-up. At the 3-month follow-up visit, only VEGF-A levels were significantly higher than in the healthy control subjects. In this explorative part of the study, a significant correlation between maximum levels of MMP-9 during hospital stay and the presence of mosaic pattern on chest CT, as well as parenchymal bands, was observed (Table 5). The levels of TIMP-2, a known inhibitor of MMP-9 activity, were not increased during the study period, compared to healthy control subjects (Fig. 3).

**Table 5:** Association of pro-fibrotic markers during hospital admission and after 3 months, with CT findings 3 months after hospital admission for COVID-19.

	Max MMP-9 Coefficient estimate (95% CI)	MMP-9 3 months Coefficient estimate (95% CI)	Max SP-D Coefficient estimate (95% CI)	SP-D 3 months Coefficient estimate (95% CI)	Max VEGF-A Coefficient estimate (95% CI)	VEGF-A 3 months Coefficient estimate (95% CI)
DL_CO_	0.25 (− 0.85, 1.35)	1.02 (0.71, 2.75)	− 0.02 (− 0.25, 0.21)	− 0.03 (0.30, 0.23)	< 0.01 (< − 0.01, < 0.01)	0.02 (< − 0.01, 0.03)
GGO	0.09 (− 0.04, 0.22)	0.05 (− 0.15, 0.25)	0.01 (− 0.02, 0.03)	0.01 (− 0.02, 0.04)	< 0.01 (< − 0.01, < 0.01)	< 0.01 (< − 0.01, < 0.01)
GGO ≥ 10%	0.16 (0.01, 0.33)*	− 0.03 (− 0.32, 0.21)	0.01 (− 0.02, 0.04)	− 0.01 (− 0.05, 0.03)	< 0.01 (< − 0.01, < 0.01)	< − 0.01(< − 0.01, < 0.01)
Mosaic	0.14 (< 0.01, 0.28)	0.24 (0.03, 0.50)*	0.02 (− 0.01, 0.05)	< 0.01 (− 0.03, 0.04)	< − 0.01 (< − 0.01, < 0.01)	< − 0.01(< − 0.01, < 0.01)
Parenchymal bands	0.19 (0.05, 0.33)**	0.258 (0.04, 0.51)*	0.02 (< − 0.01, 0.05)	0.03 (< − 0.01, 0.06)	< 0.01 (< − 0.01, < 0.01)	< − 0.01(< − 0.01, < 0.01)
Reticular pattern	0.04 (− 0.19, 0.24)	− 0.06 (− 0.49, 0.25)	0.01 (− 0.03, 0.06)	0.04 (− 0.01, 0.08)	< − 0.01 (< − 0.01, < 0.01)	< − 0.01(− 0.01, < 0.01)
Interlobular septal thickening	0.10 (− 0.07, 0.26)	− 0.36 (− 0.86, 0.01)	0.02 (− 0.01, 0.06)	0.01 (− 0.03, 0.05)	< 0.01 (< − 0.01, < 0.01)	< − 0.01(< − 0.01, < 0.01)
Consolidations	− 0.53 (− 1.62, 0.04)	− 0.01 (− 0.57, 0.35)	− 0.09 (− 0.29, 0.02)	− 0.02 (− 0.11, 0.05)	< − 0.01 (< − 0.01, < 0.01)	< 0.01(< − 0.01, < 0.01)
Bronchiectasis	0.05 (− 0.29, 0.34)	0.18 (− 0.253, 0.52)	− 0.03 (− 0.13, 0.04)	− 0.07 (− 0.24, 0.02)	< − 0.01 (< − 0.01, < 0.01)	< − 0.01(< − 0.01, < 0.01)

GGO ≥ 10%, ground-glass-opacities in ≥ 10% of ≥ 1 lung zone. Mosaic, any mosaic pattern. Parenchymal bands, any parenchymal bands. Reticular pattern, any reticular pattern. Interlobular septal thickening, any interlobular thickening. Consolidations, any consolidations. Bronchiectasis, any bronchiectasis.

*MMP9* matrix metalloproteinase 9, *SP-D* surfactant protein D, *VEGF-A* vascular endothelial growth factor A, *GGO* ground glass opacities, *CT* computed tomography, *DL*_*CO*_ in percent of predicted, *GGO* any ground-glass opacities.

*p < 0.05, **p < 0.01.

## Discussion

In this sub-study of the NOR-SOLIDARITY trial, nearly 40% of the participants reported persistent airway symptoms, and nearly 30% had DL_CO_ below LLN 3 months following hospital admission and treatment for COVID-19. Findings on chest CT were also common, with 40% presenting with GGO and a similar percentage with parenchymal bands 3 months after hospital admission. We did not find significant differences between participants randomised to remdesivir and HCQ, and their respective SoC, with regard to pulmonary function and CT-findings, except for a somewhat higher symptom burden in the remdesivir group compared to the SoC group. Of the clinical characteristics during hospitalisation, a low P/F-ratio, the need for ICU admission, a high viral load, and low levels of IgG antibodies to SARS-CoV-2 seemed to predict reduced DL_CO_% at 3 months. Finally, high plasma levels of MMP-9, both during hospital stay and 3 months after hospitalisation, were associated with signs of both reversible and irreversible changes on chest CT.

The majority of our study participants were men, and the most common comorbidities were hypertension, diabetes mellitus, and obesity, which is consistent with findings in other studies^22^. Former smoking was relatively common, but very few were current smokers. The participants had a duration of symptoms of 8 days prior to admission, and nearly 40% tested positive for SARS-CoV-2 IgG antibodies at admission, reflecting the natural course of COVID-19 infection described by others^23^. Nearly 40% of the participants presented with respiratory failure, defined by P/F-ratio < 40 kPa, and the majority had moderately elevated inflammatory markers, which is in accordance with studies from other countries^24–26^. Although previously described as a marker of COVID-19 severity, lymphopenia was not a common trait in our population at baseline^27^. Kåsine et al. recently characterised the leukocyte subpopulations in our participants, demonstrating neutrophilia as more dominant than lymphopenia during the first week of hospitalisation in our cohort, along with an association between high neutrophil counts and disease severity^28^.

In accordance with the WHO SOLIDARITY study and the NOR-SOLIDARITY sub study demonstrating no effect on in-hospital mortality, the need for ICU treatment, or P/F ratio, we show that treatment with remdesivir and HCQ for COVID-19 had no significant effect on pulmonary function test results 3 months following discharge for COVID-19, compared to SoC^5,6^. However, participants treated with remdesivir reported a higher CAT-score 3 months after hospital admission for COVID-19 than those receiving SoC.

We have previously reported decreased pulmonary function and persistent CT pathology in COVID-19 patients admitted to hospital, with results comparable to those presented in the current study^2^. However, by extending the analyses, we show a relationship between disease severity during hospital admission, as indicated by both low P/F-ratio and ICU admission, and impaired pulmonary function 3 months after discharge, as shown by reduced DL_CO_% in the fully adjusted model. The positive correlation between P/F-ratio during hospitalisation and DL_CO_% at the follow-up indicates that a reduced diffusion capacity of the lungs may persist at least 3 months after hospital admission.

Currently, it is not known how viral load affects the long-term pulmonary outcome following COVID-19. Another study found no difference in viral load between mild and severe disease in the acute phase^29^. Here we present a significant negative association between viral load in the oropharynx at hospital admission and DL_CO_% 3 months following hospitalisation. We also found a positive correlation between the presence of IgG antibodies to SARS-CoV-2 during the acute phase, and preserved diffusion capacity. Taken together, a low viral load combined with a robust and early antibody response against SARS-CoV-2 seem to protect against long-term impairment of pulmonary function. Finally, although COVID-19 seems to be characterized by sustained inflammation, plasma levels of inflammatory markers such as CRP and ferritin during hospital admission did not seem to predict a poorer pulmonary outcome at 3 months^26^.

In the present study, we also showed that nearly 40% of the participants had a CAT-score ≥ 10, which indicates a medium to high symptom burden after 3 months. The most prevalent symptoms were dyspnoea on exertion and loss of energy. Smokers are known to have a more severe course of COVID-19, and we found a higher symptom burden among participants with a history of smoking^30^. Interestingly, we found no association between CAT-score, and the pulmonary outcomes DL_CO_%, and chest CT-findings in these participants.

As an explorative outcome, we investigated the association between known pro-fibrotic mediators and the set of pulmonary outcomes. MMP-9 and VEGF-A were significantly elevated during the acute phase in these COVID-19 patients compared with healthy control subjects. From a hypothesis-generating point of view, it was interesting to see that MMP-9 levels, both during the acute phase and after 3-months, were significantly associated with persistent CT changes. Investigations have found MMP-9 in several cell types of the lung, such as alveolar macrophages, airway epithelium, and in neutrophils recruited to the lung during inflammatory processes^31,32^, but the major cellular sources of circulating MMP-9 in COVID-19 patients are still not evident. MMP-9 is involved in the complex process of extracellular matrix remodelling within the lung, and its activity is regulated by different TIMPs, including TIMP-2^33^. Increased levels of MMP-9, combined with our finding of TIMP-2 levels within normal limits throughout the observation period, suggest enhanced MMP-9 activity in these COVID-19 patients. Based on the role of MMP-9 in the inflammatory process of acute lung injury, as well as its role in the development of pulmonary fibrosis^34^, MMP-9 has been suggested as a therapeutic target in the treatment of interstitial and acute inflammatory lung diseases^35,36^. Our findings may indicate that a similar approach could be of interest in COVID-19-induced lung injury, as well.

Our cohort had significantly elevated plasma levels of VEGF-A compared to healthy control subjects, both during hospital admission and 3 months after hospitalisation. Alveolar epithelial cells are claimed to be a major source of VEGF-A in humans, and VEGF-A has for long been a mediator of interest in understanding the pathophysiology in acute lung injury and ARDS, as well as in interstitial lung diseases, like idiopathic pulmonary fibrosis (IPF)^37^. This is reflected by the fact that an existing anti-fibrotic agent for treatment of IPF is targeting VEGF-A^38^. Even though we found no association between the increased VEGF-A levels and persistent findings on chest CT, it was interesting to observe that the level of VEGF-A was still elevated 3 months after hospitalisation. Other studies have found similar results, with significantly elevated VEGF-A levels in plasma of moderate to severe COVID-19 patients^39,40^. These findings might indicate that VEGF-A plays a role in the development of COVID-19 sequelae.

Our findings are limited by a small sample size, both regarding the evaluation of treatment effects and the interpretation of sub-analyses. Although we did not find significant effects of remdesivir and HCQ, except for a negative effect of remdesivir on CAT score, we cannot firmly exclude any treatment effects. Moreover, the participants’ pulmonary status prior to inclusion was not known, and established chronic lung disease prior to COVID-19 might have influenced the results. Due to a lack of additional plasma aliquots, we were not able to pursue our findings on matrix regulating mediators by analysing MMP-9 activity, other MMPs, and additional TIMPs. Moreover, the explorative part of this study presents data of descriptive nature and we lack data on the cellular sources of MMP-9 in these COVID-19 patients. Finally, associations do not necessarily reflect a causal relationship.

## Conclusion

In this sub study of the NOR-SOLIDARITY trial, a high prevalence of respiratory symptoms, diffusion capacity impairment, and persistent chest CT-findings were observed 3 months after hospital admission for COVID-19. CAT-score seemed to be negatively influenced by treatment with remdesivir, while no difference in pulmonary function tests and CT-findings between remdesivir and HCQ, and their respective SoC group was found. A high viral load, low P/F-ratio, and admission to ICU seemed to predict reduced diffusion capacity in the lungs at 3 months, whereas high antibody levels at baseline had a positive effect on pulmonary function. The association between the pro-fibrotic mediator MMP-9 and persistent CT-findings highlights the need for more knowledge about the development of pulmonary fibrosis following hospitalisation for COVID-19.

## Supplementary Information

Supplementary Tables.

Supplementary Information.

## Data Availability

De-identified patient level data for participants in this study, statistical analysis plan and statistical coding can be made available after the approval of the institutional review board. Requests should be made to the corresponding author.
