# Supplementary material for: Persistent pulmonary pathology after COVID-19 is associated with high viral load, weak antibody response, and high levels of matrix metalloproteinase-9
Source: Sci Rep. 2021 Dec 1;11:23205. doi: 10.1038/s41598-021-02547-x (PMC8636497; doi:10.1038/s41598-021-02547-x)
Supplement: Supplementary file 1 — Supplementary Tables. [file 41598_2021_2547_MOESM1_ESM.pdf]

Supplementary table 1. Overview of missing values, n (%).

	**Overall, n = 108**	**Remdesivir + SoC, n= 16**	**SoC, n=27**	**HCQ + SoC, n = 40**	**SoC, n = 47**
**Demographics**					
Age, years	0	0	0	0	0
Female, n (%)	0	0	0	0	0
Body Mass Index (kg/m^2)	6 (5.6)	0	2 (7.4)	2 (5.0)	4 (8.5)
Smoking history	4 (3.7)	2 (12.5)	1 (3.7)	0 (0)	2 (4.3)
**Comorbidities**					
Chronic pulmonary disease	0	0	0	0	0
Hypertension	0	0	0	0	0
Diabetes mellitus	0	0	0	0	0
Obesity (BMI > 30 kg/m^2), n (%)	6 (5.6)	0	2 (7.4)	4 (8.5)	2 (5.0)
**Baseline characteristics**					
Duration of symptoms prior to baseline (days)	0	0	0	0	0
P/F-ratio (kPa)	1 (0.9)	0	0	1 (2.5)	0
Respiration rate per minute	0	0	0	0	0
Temperature (°C)	0	0	0	0	0
Admission to ward, n (%)	0	0	0	0	0
Admission to ICU, n (%)	0	0	0	0	0
**Biochemical presentation**					
Haemoglobin (g/dL)	0	0	0	0	0
White blood cell count (x 10^9 per L)	0	0	0	0	0
Lymphocytes (x 10^9 per L)	5 (4.6)	0	2 (7.4)	2 (5.0)	2 (4.3)
Platelet count (x 10^9 per L)	0	0	0	0	0
Creatinine (μmol/L)	1 (0.9)	0	0	1 (2.5)	0
Ferritin (μg/L)	4 (3.7)	0	0	1 (2.5)	3 (6.4)
CRP	0	0	0	0	0

LDH (U/L)	3 (2.8)	0	2 (7.4)	0	3 (6.4)
**Viral load (oropharynx) at baseline**	25 (23.1)	3 (18.8)	2 (7.4)	13 (32.5)	9 (19.1)
**Anti-SARS-CoV-2 antibodies at baseline**	26 (24.1)	2 (12.5)	7 (25.9)	11 (27.5)	12 (25.5)
**Spirometry at three months**	7 (6.5)	1 (6.2)	0	2 (5.0)	4 (8.5)
**Diffusion capacity at three months**	8 (7.4)	1 (6.2)	0	3 (7.5)	4 (8.5)
**CAT-score at three months**	4 (3.7)	1 (6.2)	0	2 (5.9)	1 (2.1)
**Chest CT at three months**	6 (5.6)	0	1 (3.7)	3 (7.5)	2 (4.3)
**Biobanking during hospital admission**	17 (15.7)	6 (23.0)		4 (12.5)	
**Biobanking at three months**	10 (9.3)	3 (11.5)		3 (9.4)	

SoC, standard of care. HCQ, hydroxychloroquine. BMI, body mass index. P/F ratio, arterial oxygen pressure (pO_2) divided by fraction of inspired oxygen (f_iO_2). ICU, intensive care unit. CRP, C-reactive protein. LDH, lactate dehydrogenase. CAT-score, COPD assessment test score. CT, computed tomography.

Supplementary Table 2. Association between symptoms assessed by CAT-score, DL_{CO}%, and chest CT findings compared with normal chest CT at three months after hospital admission for COVID-19.

**Respiratory symptoms (CAT score > 10)**	**Coefficient estimate**	**95 % confidence interval**
DL_{CO}%	< -0.01	(-0.03, 0.02)
Reversible CT changes (any)	-0.58	(-2.02, 0.71)
Irreversible CT findings (any)	0.43	(-0.41, 1.30)

CAT-score, the COPD assessment test score. $DL_{CO,}$ diffusion capacity of the lung for carbon monoxide, in percent of predicted. CT, computed tomography. Reversible CT findings defined as any occurrence of ground-glass opacities or mosaic pattern on chest CT. Irreversible CT findings defined as any occurrence of parenchymal bands, reticular pattern, interlobular septal thickening, consolidations or bronchiectasis.
