# Supplementary material for: Persistent pulmonary pathology after COVID-19 is associated with high viral load, weak antibody response, and high levels of matrix metalloproteinase-9
Source: Sci Rep. 2021 Dec 1;11:23205. doi: 10.1038/s41598-021-02547-x (PMC8636497; doi:10.1038/s41598-021-02547-x)
Supplement: Supplementary file 2 — Supplementary Information. [file 41598_2021_2547_MOESM2_ESM.pdf]

The Investigational WHO NOR-COVID 19 protocol

Project title
The NOR Solidarity multicenter trial on the efficacy of different anti-viral drugs in SARS-CoV-2 infected patients

Short title: The efficacy of different anti-viral drugs in SARS-CoV-2

EudraCT no: 2020-000982-18
Version: 7 2020-03-30

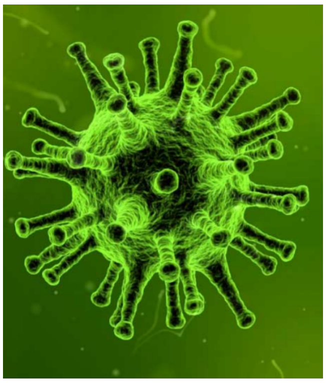

CONFIDENTIAL
This document is confidential and the property of the study group. No part of it may be transmitted, reproduced, published, or used without prior written authorisation.

STATEMENT OF COMPLIANCE
This document is a protocol for a clinical research study. The study will be conducted in compliance with all stipulations of this protocol, the conditions of Regional Ethic committee approval and the latest version of the Helsinki Declaration.

This protocol describes a multicenter add-on trial that is part of and based on the global WHO COVID-19 core protocol. The aim of the WHO COVID-19 core protocol is to evaluate and compare four different anti-viral treatment regimens on in-hospital mortality in moderate and severe COVID infected patients. This multicenter add-on trial will be a separate and independent trial but will use the global level randomization and contribute to report endpoints and adverse events to the global trial.

Synopsis of the protocol

Terminology: The novel coronavirus-induced disease first described in 2019 in China is designated COVID-19 (or COVID), and the pathogen itself (an RNA virus) is SARS-coronavirus-2 (SARS-CoV-2). The Norwegian study that is an adapted WHO-study and follows the WHO protocol whilst also analyzing additional factors is referred to as WHO NOR-COVID 19 study. Standard of Care will be referred to as SoC.

Background: In early 2020 there were no approved anti-viral treatments for COVID, and WHO expert groups advised that four re-purposed drugs, Hydroxychloroquine, Remdesivir, Lopinavir with Ritonavir and Interferon (β1a) should be evaluated in an international five-arm randomized trial where the fifth arm is SoC. The WHO NOR-COVID 19 study will start analyzing the effects of Hydroxychloroquine and Remdesivir initially and may open for a fourth arm.

Patient enrolment and randomization: This will be carried out online via a database created for this purpose by the WHO. Adults (age ≥18 years) recently hospitalized, or already in hospital, with definite COVID and, in the view of the responsible doctor, no contra-indication to any of the study drugs will be randomly allocated between

- Local SoC alone,

OR local SoC plus one of

- Remdesivir
- Hydroxychloroquine

Data reported before randomization via the WHO database:

- Hospital and randomizing doctor
- Confirmation that informed consent has been obtained
- Patient identifiers, age and sex
- Patient characteristics (yes/no): current smoking, diabetes, heart disease, chronic lung disease, chronic liver disease, asthma, HIV infection, active tuberculosis.
- COVID-19 severity at entry (yes/no): shortness of breath, being given oxygen, already on a ventilator, and, if lungs imaged, major bilateral abnormality (infiltrations/patchy shadowing)
- Whether any of the study drugs are currently NOT AVAILABLE at the hospital.

Inclusion criteria

- Adult patients, 18 years and older
- Confirmed SARS 2 CoV 2 infection by PCR
- Admitted to the hospital ward or the ICU
- Subjects (or legally authorized representative) provide written informed consent prior to initiation of the study
- No anticipated transfer within 72 hours to a non-study hospital

Exclusion criteria

- Severe co-morbidity with life expectancy <3 months according to investigators assessment
- ASAT/ALAT > 5 times the upper limit of normal
- Acute co-morbidity within 7 days before inclusion such as myocardial infarction
- Known intolerance to the available study drugs
- Pregnancy or breast feeding
- Any reason why, in the opinion of the investigators, the patient should not participate
- Subject participates in a potentially confounding drug or device trial during the course of the study
- Prolonged QT interval (>470 ms)

Drug safety: Suspected unexpected serious adverse reactions that are life-threatening (e.g. anaphylaxis, aplastic anemia, or anything comparably uncommon and serious) must be reported within 24 hours of being diagnosed, without waiting for death or discharge.
Outcomes: The primary outcome is all-cause mortality, subdivided by severity of disease at the time of randomization. The major secondary outcomes are duration of hospital stay, time to first receiving ventilation (or intensive care), viral clearance, kidney failure, myocardial failure, co-infections, organ dysfunction, quality of life after 3 months, Inflammatory and anti-inflammatory mediators, markers of extracellular matrix remodeling, markers of endothelial activation and markers of platelet activation.

Data monitoring: A Data and Safety Monitoring Committee will keep the accumulating drug safety results and major outcome results under regular review.
Adaptive design: The WHO may decide to add novel treatment arms while the trial is in progress. Conversely, the WHO may decide to discontinue some treatment arms, especially if the Global Data and Safety Monitoring Committee reports, based on interim analyses, that one of the trial treatments definitely affects mortality.
Data security: Patient information will be encrypted and held securely by the WHO and sponsor. Those analyzing it will use only anonymized data, and no identifiable patient details will appear in publications.

Table of Contents

1 Investigators and facilities 7
1.1 Study locations and principal investigators 7
1.2 Study Management 8
1.3 Monitor 8

2. Background and objectives 9
2.1 Background Information 9
2.1.1 Clinical trial rational 9
2.2 Target audience 9
2.3 Study design 10

3 Objectives and endpoints 12

4 Study population 13
4.1 Inclusion criteria (as described in WHO COVID 19 core protocol) 13
4.2 Exclusion criteria 13
4.3 Baseline characteristics 13

5 Study products and study drug regimes 13
5.1 Remdesivir 13
5.2 Hydroxychloroquine 14
5.3 Medical side effects and management of Remdesivir 14
5.4 Medical side effects and management of Hydroxychloroquine 14
5.5 General information on intervention drugs 14

6 Drug discontinuation and patient withdrawal 15

7 Study assessments and procedures 15
7.1 Clinical variables 15

7.2 Case report form and patient numbers 15
7.3 Risks to participants 16
7.4 Benefits to participants 16

7.5 Specimens and laboratory analysis 16

8 Data management 17

9 Safety monitoring and reporting 17
9.1 Adverse Events and Serious Adverse Events 17
9.1.1 Definition of Adverse Event (AE) 17
9.1.2 Definition of Serious Adverse Event (SAE) 18
9.1.3 Suspected Unexpected Serious Adverse Reactions (SUSAR) 18
9.1.4 Classification of an Adverse Event 18
9.1.5 Severity of Adverse Events 18
9.1.6 Relationship to Study Intervention 19
9.1.7 Time Period and Frequency for Event Assessment and Follow-Up 19
9.1.8 Investigators Reporting of AEs 19
9.1.9 Serious Adverse Event Reporting 19
9.2 Procedures in Case of Emergency 20
9.3 Safety Committee 20
9.4 Protocol deviations 20

10 Statistical methods and data analysis 20
10.1 Determination of Sample Size 20

10.2 Randomization 20
10.2.1 Allocation- sequence generation 20
10.2.2 Allocation- procedure to randomize a patient 20
10.2.3 Blinding and emergency unblinding 21
10.3 Population for Analysis 21
The primary population is the ITT population. 21
10.4 Planned analyses 21
10.5 Statistical Analysis 21
10.5.1 Primary analysis 21
10.5.2 Secondary analyses 22
10.5.3 Safety analyses 22
10.5.4 Descriptive statistics 22
10.5.5 Missing data 22

11 Ethical considerations 22
11.1 Informed Consent 23
11.2 Confidentiality 24

12 Scientific and peer review 24

13 References 25

Appendix 26
A1 The WHO COVID-19 core protocol, version 10 26
A2 WHO Standard Operating Procedures and appendix, version 10 26
A3 National early warning score 26
A4 Sofa Score 26
A4 Clinical Frailty Scale 27
A5 Preparation of plasma and serum (study samples) 27
A6 Participating hospitals and contact information 28
A7 Flow chart 28

STATEMENT OF COMPLIANCE

The study will be carried out in accordance with the following, as applicable:
All National and Local Regulations and Guidance applicable at each site The International Council for Harmonisation of Technical Requirements for Registration of Pharmaceuticals for Human Use (ICH) E6 (R2) Good Clinical Practice, and the Belmont Report: Ethical Principles and Guidelines for the Protection of Human Subjects of Research, National and ethical regulations

The signature below provides the necessary assurance that this study will be conducted according to all stipulations of the protocol including statements regarding confidentiality, and according to local legal and regulatory requirements, and ICH E6(R2) GCP guidelines.

Site Investigator Signature:

____________________________ ____________________________

Signed: Date:

Name:

Title:

1 Investigators and facilities

1.1 Study locations and principal investigators

Study sites will include hospitals governed by different regional authorities and have one or two principal investigators dependent on the size of the hospital. As of today, 22 different hospital sites are participating. Contact information is to be found in Appendix 6.

	PI
**Northern Norway Regional Health Authority**	
• University Hospital of North Norway, Tromsø (UNN)	Anders Benjamin Kildal<br>Vegard Skogen
• Nordland Hospital, Bodø	Hanne Winge Kvarenes
**Central Norway Regional Health Authority**	
• St. Olav University Hospital, Trondheim	Pål Klepstad<br>Raisa Hannula
• Levanger Hospital	Nina Vibeche Skei
• Molde Hospital	Birgitte Tholin
• Ålesund Hospital	Dag Arne Lihaug Hoff
**Western Norway Regional Health Authority**	
• Haukeland University Hospital, Bergen	Bjørn Blomberg<br>Reidar Kvåle
• Haraldsplass Hospital, Bergen	Bård Reiakvam Kittang
• Stavanger University Hospital	Åse Berg
**South-Eastern Norway Regional Health Authority**	
• Akershus University Hospital	Olav Dalgård
• Vesfold Hospital, Tønsberg	Asgeir Johannessen
• Telemark Hospital, Skien	Hilde Kristin Skuedal
• Østfold Hospital, Kalnes	Waleed Ghanima
• Kristiansand Hospital	Mette Haugli
• Arendal Hospital	Roy Bjørkholt Olsen
• Drammen Hospital	Lars Heggelund
• Bærum Hospital	Anders Aune Tveita
• Ringerike Hospital	Lars Thoresen
• Kongsberg Hospital	Gernot Ernst
• Oslo University Hospital (OUH)	
○ Ullevål	Anne Margarita Dyrhol Riise<br>Aleksander Rygh Holten
○ Rikshospitalet	Pål Aukrust<br>Andreas Barratt-Due
• Diakonhjemmet Hospital, Oslo	Leif Erik Vinge
• Lovisenberg Hospital, Oslo	Hedda Hoel
• Innlandet Hospital Trust	Ragnhild Eiken

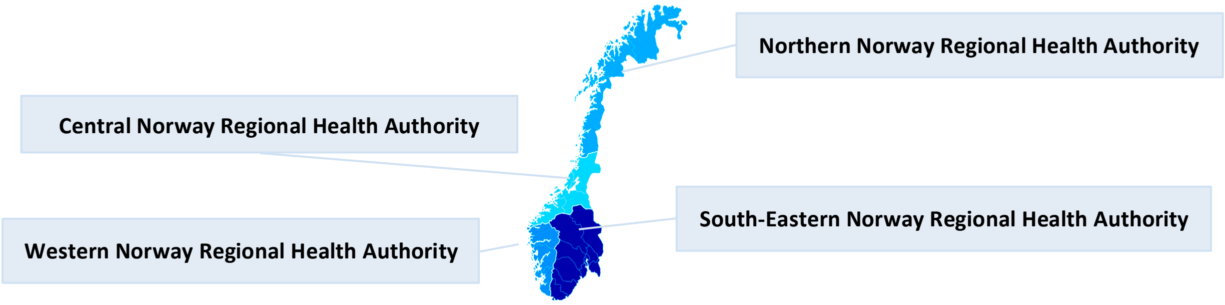

Figure 1: Norway divided into four regional Health Authorities

1.2 Study Management

The Steering and Management Committee will manage and coordinate the study centrally. As of today the steering committee is composed of:

Project leader: Pål Aukrust, MD, PhD, Professor; OUH Rikshospitalet
Andreas Barratt-Due, MD, PhD; OUH Rikshospitalet
Trine Kåsine, MD; OUH Rikshospitalet
Katerina Nezvalova-Henriksen, Cand. Pharm, PhD; OUH Rikshospitalet
Anne Margarita Dyrhol Riise, MD, PhD, Professor; OUH Ullevål
Marius Trøseid, MD, PhD; OUH Rikshospitalet
Inge Christoffer Olsen, Statistician, PhD, NorCRIN

Locally, the study will be managed and coordinated by a Principal Investigator, with responsibility for data collection and maintenance of study documentation.

1.3 Monitor

The study will be monitored by clinical study monitors located at the University Hospital of North Norway, St Olav's Hospital, Haukeland University Hospital and Oslo University Hospital.

All sites will be visited and/or contacted by phone on a regular basis by the monitor, who will verify that informed consent process, reporting of adverse events and other safety data, adherence to protocol, maintenance of required regulatory documents, facilities and equipment and data completion on the case report forms, including source data verification are up to standard. The details of the monitoring, e.g. the processes, equipment and data to be checked, will be described in a study specific monitoring plan.

2. Background and objectives

2.1 Background Information

Infectious diseases are the single biggest cause of death worldwide. New infectious agents, such as the SARS, MERS and other novel coronavirus as well as influenza viruses, all represent a threat and disease burden to the society and health facilities. Influenza epidemics and pandemics worldwide continue to challenge public health and health care systems. The pandemic in 2009-2010 resulted in 100.000-400.000 deaths, affecting not only elderly individuals and individuals with co-morbidities, but also children and young adults (1). The present pandemic with COVID-19 is expected to result in a high number of critically ill patients in need of hospitalization and respiratory support, and an unknown proportion of patients who inevitably will die. As of today, no specific treatment of COVID-19 has been established. To this end, Remdesivir is the only treatment option that directly inhibits SARS-CoV-2 RNA polymerase and has been shown to inhibit virus replication in vitro. It has also been shown to have protective effects against the familiar coronavirus MERS (2), and notably, both preclinical experiments and case reports suggest that Remdesivir could have beneficial effects in COVID 19 (3, 4). Another promising treatment option is hydroxychloroquine which seems to be effective in limiting the replication of SARS-CoV-2 *in vitro* at least partly by interfering with the pH-dependent endosome-mediated viral entry, and its potential effect in COVID-19 has been suggested by six publications and ongoing clinical trials (5). Clinical experience with the use of neuraminidase inhibitors demonstrated that prompt treatment probably represents the most effective strategy for the management of influenza epidemics (6). It is conceivable that a similar early treatment strategy is also necessary in COVID 19 patients and is applicable to other drug therapies, but so far, no data exist to support this assumption.

The WHO COVID 19 core protocol has been designed to prospectively collect clinical data globally, and rapidly evaluate and conclude whether different anti-viral treatment regimens can reduce in-hospital mortality. Along with this, separate and independent add-on trials will use the global level randomization centre and contribute to report endpoints and adverse events as encouraged by the WHO (7).

2.1.1 Clinical trial rationale

The rationale for the study is to test whether available anti-viral drugs with the potential to inhibit SARS-CoV-2 (i.e. Hydroxychloroquine and Remdesivir) have any clinical effect on COVID-19 infected patients.

Knowledge from this study will hopefully contribute to the clarification of whether Remdesivir and Hydroxychloroquine are beneficial or not and if early Remdesivir and Hydroxychloroquine intervention is needed to have any beneficial effects. This is important knowledge and highly relevant for future treatment of COVID-19 infected patients.

Data from the study will also provide increasing knowledge of the pathogenesis of severe COVID-19 infection and in particular, which molecular pathways and biomarkers that characterize those patients that need ICU management and those that could be managed at the hospital ward.

2.2 Target audience

Departments of Infectious Diseases and ICU's at University- and larger hospitals in Norway treating COVID-19 infected patients are invited to participate in this study and contribute with data to a centralized database. We encourage all centres to contribute to this effort. In all cases, a proportionate case report form (a web-based electronic "eCRF") will be completed.

2.3 Study design

The WHO NOR-COVID-19 study is an adaptive, randomized, open clinical trial to evaluate the safety and efficacy of possible therapeutic agents in hospitalized adult patients diagnosed with COVID-19. See the WHO COVID-19 core protocol (Appendix 1) for the core trial design. This multicentre add-on trial will follow the WHO core protocol version 10 dated 22.03.2020 but will add additional secondary and exploratory endpoints.

Adults (age ≥18 years) recently hospitalised, or already in hospital, with definite COVID and, in the view of the responsible doctor, no contra-indication to any of the study drugs will be randomly allocated between

- Local standard of care alone,

OR local standard of care plus one of

- Remdesivir (daily infusion for 10 days)
- Hydroxychloroquine (two oral loading doses, then orally twice daily for 10 days)

The primary objective of the trial is to investigate the effect of Remdesivir and Hydroxychloroquine on all-cause in-hospital mortality.

It was decided to conduct an open trial as preparing placebo for the two treatment groups was not feasible to start rapidly. It is also complex to handle in a trial with 2 active treatment groups and if additional arms might be added.

Patients will be followed daily during hospitalisation, and then by telephone after 3 months or outpatient clinical visit.

In the core protocol follow-up data are only collected at discharge. In this protocol subjects will additionally be assessed for efficacy and safety both during hospitalisation and after hospitalisation.

In addition to the global independent data and safety monitoring board (DSMB) as described in the core protocol, a national DSMB will monitor the safety and risk-benefit of the of the trial interventions in the Norwegian population.

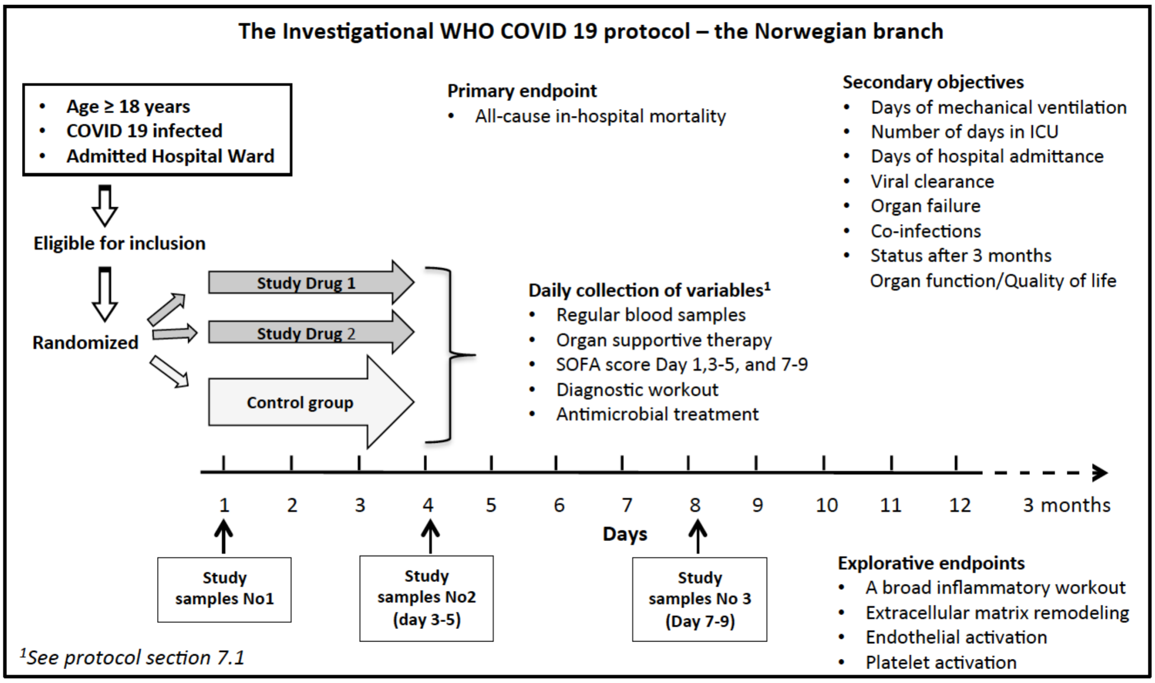

Figure 2: The WHO NOR-COVID 19 protocol

3 Objectives and endpoints

Objectives	Endpoints (Outcome measures)
**Primary** (in line with WHO core protocol)	
To investigate the effect of Remdesivir and Hydroxychloroquine on all-cause in-hospital mortality in moderate and in severe COVID.	All-cause in-hospital mortality
**Secondary** (in line with WHO core protocol)	
**Assess the** effect of Remdesivir and Hydroxychloroquine treatment on hospital duration, receipt of ventilation or intensive care, and to identify any serious adverse reactions.	During hospitalisation<br>• Receipt of mechanical ventilation<br>• Time to first receiving and duration of mechanical ventilation<br>• Receipt of intensive care<br>• Duration of intensive care<br>• Occurrence of Suspected Unexpected Serious Adverse Reactions (SUSARs)
**Secondary and exploratory** (as addition to the WHO core protocol)	
**Assess** the effect of Remdesivir and Hydroxychloroquine on 28 days mortality, viral clearance, kidney and myocardial failure, co-infections, organ dysfunction, health-related Quality of Life and biomarkers	During hospitalisation<br>• Viral clearance as assessed by SARS-CoV-2 PCR in peripheral blood and nasopharyngeal and lower airway specimens during hospitalisation<br>• Occurrence of kidney failure (eGFR <40)<br>• Occurrence of myocardial failure (left ventricular ejection fraction <40 % as assessed by echocardiography)<br>• Occurrence of co-infections (super infections with bacteria, fungi and other virus)<br><br>Assessed during or after 3 months<br>• Presence of organ dysfunction (i.e., pulmonary, renal, cardiac and cerebral) after 3 months<br>• Quality of life after 3 months assessed by version 1.2 of the generic 36-item Short Form Health Survey (SF-36) and the 5-dimension EuroQol (EQ-5D) questionnaires.<br>• 28 days mortality<br><br>Biomarkers during hospitalisation<br>• Inflammatory and anti-inflammatory mediators as assessed in serum and plasma<br>• Markers of extracellular matrix remodelling<br>• Markers of endothelial activation<br>• Markers of platelet activation
Further assess safety of Remdesivir and Hydroxychloroquine on severe, life-threatening and /or serious adverse events	Adverse events during hospitalisation

4 Study population

4.1 Inclusion criteria (as described in WHO COVID 19 core protocol)

1. Adult patients, 18 years and above
2. Confirmed SARS-2-CoV-2 infection by PCR
3. Admitted to the hospital ward or the ICU
4. Subjects (or legally authorized representative) provides written informed consent prior to initiation of the study
5. No anticipated transfer within 72 hours to a non-study hospital

4.2 Exclusion criteria

1. Severe co-morbidity with life expectancy <3 months according to investigators assessment
2. ASAT/ALAT > 5 times the upper limit of normal
3. Acute co-morbidity within 7 days before inclusion such as myocardial infarction
4. Known intolerance to the available study drugs
5. Pregnancy or breast feeding
6. Any reason why, in the opinion of the investigators, the patient should not participate
7. Subject participates in a potentially confounding drug or device trial during the course of the study
8. Prolonged QTc interval (>470 ms)
9. Already receiving any of the study drugs
10. Hydroxychloroquine should not be given to patients with psoriasis

4.3 Baseline characteristics

According to the CRF a description of the included patients co-morbidity, ongoing treatment and performance status (Frailty score) will be required. Severe disease is defined as patients which need ICU management and mild/moderate disease is defined as people which can be treated at the hospital ward.

A pregnancy test will be performed in all women of childbearing potential. For the purpose of this document, a woman is considered of childbearing potential (WOCBP), i.e. fertile, following menarche and until becoming post-menopausal unless permanently sterile. Permanent sterilisation methods include hysterectomy, bilateral salpingectomy and bilateral oophorectomy

5 Study products and study drug regimes

See core protocol (Appendix 1 and SOP-4 to SOP-7b). Note that this protocol currently only includes hydroxychloroquine and remdesivir as study drugs.

5.1 Remdesivir

The lyophilized formulation of Remdesivir is a preservative-free, white to off-white or yellow, lyophilized solid containing 100 mg of Remdesivir to be reconstituted with sterile water for injection and diluted into IV infusion fluids prior to IV infusion. It is supplied as a sterile product in a single-use, 50 mL, Type 1 clear glass vial. In addition to the active ingredient, the lyophilized formulation of Remdesivir contains the following inactive ingredients: water for injection, SBECD (sulfobutylether cyclodextrin), hydrochloric acid, and/or sodium hydroxide. Hydrochloric acid and/or sodium hydroxide are used to adjust the formulation to a final pH of 3.0 to 4.0 following reconstitution.

Remdesivir will be delivered by WHO when available. Remdesivir will be given intravenously with a loading dose of 200 mg at inclusion, day 1. Thereafter the patient will receive 100 mg intravenously from day 2 to day 10.

The total volume of administration can be 250 mL or 500 mL of Normal Saline. The infusion can be administered over a period of between 30 minutes and 2 hours.

5.2 Hydroxychloroquine

Hydroxychloroquine will be given orally (in the ICU in gastrointestinal tubes) with 800 mg x 2 loading dose (4 tablets x 2) followed by 400 mg x 2 (2 tablets x 2) every day for 10 days. The two loading doses are scheduled to be given 6 hours apart. The maintenance doses are scheduled to begin 6 hours after the second loading dose and to be given 12 hours apart. Hence, the final maintenance dose is scheduled to be given 10 days after the first loading dose.

5.3 Medical side effects and management of Remdesivir

In clinical studies, no evidence of nephrotoxicity has been observed with doses of Remdesivir up to 225 mg or multiple doses of 150 mg for up to 14 days.
Remdesivir should not be used with drugs that have potential hepatotoxicity. Remdesivir is pro-drug that is metabolized to its active drug through P450 3A4 (CYP-3A4) and other medications that influence the activity of this enzyme should be avoided.

Except for the intervention with Remdesivir all medical management will be provided according to standard of care at the treating site. The Research intervention with Remdesivir may potentially induce adverse effects most likely to be hypotension (8)

Specific attentions to this potentially related side effect will therefore be followed closely.

5.4 Medical side effects and management of Hydroxychloroquine

The Research intervention with Hydroxychloroquine may potentially induce adverse effects most likely to be:

- QT-prolongation
- Retinopathy (seen after long (weeks/months) exposure)
- Blood glucose fluctuations
- Haemolysis in G6PD-deficiency (very rare in the Norwegian population and therapy can start whilst suspected patients are being tested) (9)

A corrected QT interval (QTc) >470ms excludes the patient from participating in the study. If QTc increases to ≥ 500ms hydroxychloroquine must be withdrawn immediately. Additionally, ECG monitoring is needed if used together with other drugs that induce QT prolongation. Hydroxychloroquine should not be used together with antacids and - separate administration by about 4 hours is advised (9) and. Patients with diabetes treated with insulin and/or other anti-diabetic regimens will carefully monitored for hypoglycaemia. The doses of anti-diabetic treatment will be reduced according to blood sugar level. Dose-adjustment if sever kidney failure (eGFR < 10mL/min) to 50% to full dosage. No dose-adjustment when the patient is receiving continuous renal replacement therapy. The patients will be informed about the risk of developing hypoglycaemia upon treatment with hydroxychloroquine including the signs, symptoms and treatment options.

5.5 General information on intervention drugs

Hydroxychloroquine and Remdesivir are both metabolized by the P450 metabolic pathway, namely the CYP 2C8, 2D6, 3A4 enzymes. Considering extensive interpatient variation regarding the expression of these enzymes and also achievement of desirable plasma levels of these drugs, we will focus on recording the co-administration of known strong inducers of these enzymes as it is these that may influence the availability of these drugs to a clinically significant level. This

information will be used to adjust for potential confounding. The drugs in question are dexamethasone, haloperidol, carbamazepine, phenytoin, rifampin and phenobarbital.

Information about the administration, preparation, doses, storage and handling, disposition of unused product and the maintenance of inventory logs is provided in the Pharmacy manuals for Remdesivir (SOP-4) and Hydroxychloroquine (SOP-7).

6 Drug discontinuation and patient withdrawal

As described in the core protocol (Appendix 1):

At all times the patient's medical team remains solely responsible for decisions about that patient's care and safety. Hence, study drug administration must be stopped if the team suspects any serious unexpected drug-related reaction that is life-threatening. The study will be stopped in case of occurrence of AEs unknown to date in respect to their nature, severity, and duration that may negatively affect the benefit/risk of the trial.

Patients are free to withdraw from study treatment at any time, but could still remain in the study, with in-hospital outcome reported to the study at death or discharge.

Patients are also free to withdraw from the whole study at any time without any consequence and would continue to be offered the local standard of care (but not be reported on). When patients cannot withdraw from study treatment or the whole study themselves, due to their current health status, an appropriate patient representative can withdraw them on their behalf

7 Study assessments and procedures

See core protocol (Appendix 1) for core assessments.

In addition the following will be assessed for this national add-on protocol (see flow chart).

7.1 Clinical variables

Collecting of daily clinical data will start when the patient is included in the study and be continued for up to 14 days after enrolment. The following data will be collected (See, CRF for detailed description)

- Regular blood samples (see above).
- Respiratory support
- Other organ supportive support therapy (e.g., circulatory, kidney)
- SOFA score at admittance and day 3-5, 7-9
- Blood pressure, heart rate, respiratory rate, oxygen tension and temperature (for patients at hospital ward/not ICU)
- Diagnostic workout
- Co-infections
- Antimicrobial treatment in addition to the study drug
- Chest X-ray or CT scan

Samples required for clinical management will at all times have priority over samples taken for research tests. Aliquots or samples for research purposes should never compromise the quality or quantity of samples required for medical management. Wherever practical, taking research samples should be timed to coincide with clinical sampling.

7.2 Case report form and patient numbers

There will be two electronic web-based case report forms (eCRFs) for data entering. The core data collection and randomisation will be done in the WHO eCRF (see Appendix 1 for details), while national-specific data according to this protocol will be collected in a separate eCRF. The

patient study number issued from the WHO eCRF will be registered in the national eCRF for validity.

The local study site will keep a person-identifiable code secured at a separate place, available only for the primary local investigators. The study samples taken at baseline, day 1, day 3-5, day 7-9, thereafter weekly or when discharged from hospital/ICU, will be assigned a corresponding number to that of the patient as well as a number indicating sample order (1=baseline, 3=day 3).

7.3 Risks to participants

7.3.1 Remdesivir
Risks associated with Remdesivir treatment are expected to be hypotension (8), transient elevation in transaminases and transiently increased respiratory rate (personal communication with the manufacture).

7.3.2 Risk associated with Hydroxychloroquine treatment are expected to be blood glucose fluctuations, QT-prolonagtion and retinopathy (9). However, retinopathy is very unlikely to occur after only 5-10 days therapy.

7.3.3 General risk considerations
The patients included in this study will be severely ill, possibly in need of organ supportive care, and the implementation of a new drug in this situation may induce unknown effects that may aggravate organ dysfunction. Thus, Remdesivir and Hydroxychloroquine must be provided under special vigilance. On the other hand patients with COVID-19 infection at ICU will have a high mortality rate and without any established anti-viral therapy, the potential beneficial effects of this promising anti-viral agent will in our opinion outweigh the potential adverse effects of drugs in this severely ill patient group.

Blood sampling: This represents an insignificant inconvenience for the included patients. Blood samples will be taken at baseline (day 1) and day 3-5, and will constitute a minimal blood loss and no harm for the patients.

7.4 Benefits to participants

7.4.1 Remdesivir
We anticipate that intravenous administration of this drug will be particularly beneficial for patients admitted to ICU.

7.4.2 Hydroxychloroquine
This is an established drug with a well documented safety profile.

7.5 Specimens and laboratory analysis
Routine medical biochemistry data will be collected daily at each centre and analyzed by the local laboratory.

Samples for SARS-CoV-2 PCR analyses will be analysed in peripheral blood as well as from the nasopharyngeal airway at admission and every third day. When the patient is intubated, parallel samples from the lower respiratory tract will also be analyzed.

In addition, blood study samples will be taken at inclusion of the study (baseline) and at day 4 (3-5) and day 8 (7-9), thereafter weekly and/or when discharged ICU/hospital. At each time point it will be taken: (i) EDTA, 12mL and (ii) Serum 8mL. Additionally, a few study sites will take whole blood EDTA samples (4 mLs) 8 times during the study. It might be difficult for all participating

centers to collect these samples therefore lack of collection will not be considered as a protocol deviation.

Samples will be processed and centrifuged within 1 hour and stored at -80°C before analysed collectively at Research Institute of internal medicine, and Department of Immunology, Oslo University Hospital. We recognize that -80°C storage is not available at all sites. In this case it will be possible to store the samples temporarily at -20°C for 1 month before transfer to the reference laboratory. For preparation of plasma and serum, see also appendix A5.

Strict Biosafety procedures and international regulations will be applied with regard to the collection, storage, transfer and laboratory handling of research samples. Appropriate processing and storage of samples will be carefully reviewed when different hospitals and study sites decide to adhere to the study.

Research Institute of Internal Medicine at Oslo University hospital has extensive experience in analysing relevant markers in a large number of samples using multiplex technology, enzyme immune assays on a robot platform and proteomics.

8 Data management

International data management will be performed by the WHO (see Appendix 1), while national data management will be performed at Oslo University Hospital. Patients' identities will be protected and their information held securely. Only anonymized data will be stored at the web databases, whereas the participant list will be stored separately, secured, at the different local study sites.

It is important that data generated now are not destroyed unnecessarily, since they will be of considerable potential value to future generations faced with similar outbreaks of infectious disease. Electronic data and electronic copies of paper documents will be stored for at least 15 years.

9 Safety monitoring and reporting

The PI is responsible for the detection and documentation of events meeting the criteria and definition of an adverse event (AE) and a serious adverse event (SAE). The sponsor is responsible for assessing whether a SAE is defined as SUSAR as well as for reporting of all SUSARs to the national competent authority. Each patient will be instructed to contact the investigator immediately should they manifest any signs or symptoms they perceive as serious. Safety analyses will include tabulation of the type and frequency of adverse events. Any serious adverse events will be reported with comprehensive narratives. Any value of safety laboratory parameters outside the expected ranges will be identified. The methods for collection of safety data are described below.

9.1 Adverse Events and Serious Adverse Events

9.1.1 Definition of Adverse Event (AE)

AE means any untoward medical occurrence associated with the use of an intervention in humans, whether or not considered intervention related. An AE can therefore be any unfavorable and unintended sign (including an abnormal laboratory finding), symptom, or disease temporally associated with the use of medicinal (investigational) product.

Any medical condition that is present at the time that the subject is screened will be considered as baseline and not reported as an AE. However, if the severity of any pre-existing medical condition increases, it should be recorded as an AE.

Given the nature of severity of the underlying illness, subjects will have many symptoms and abnormalities in vitals and laboratory. All Grade 3 AEs will be captured as AEs in this trial and will be considered as notable events.

9.1.2 Definition of Serious Adverse Event (SAE)
An SAE is defined as "An AE or suspected adverse reaction is considered serious if, in the view of either the investigator or the sponsor, it results in any of the following outcomes:

- Death,
- a life-threatening AE,
- prolongation of existing hospitalization,
- a persistent or significant incapacity or substantial disruption of the ability to conduct normal life functions,
- or a congenital anomaly/birth defect.
- important medical events

Important medical events that may not result in death, be life-threatening, or require hospitalization may be considered serious when, based upon appropriate medical judgment, they may jeopardize the patient or subject and may require medical or surgical intervention to prevent one of the outcomes listed in this definition

"Life-threatening" refers to an AE that at occurrence represents an immediate risk of death to a subject. An event that may cause death if it occurs in a more severe form is not considered life-threatening. Similarly, a hospital admission for an elective procedure is not considered a SAE. All SAEs, as with any AE, will be assessed for severity and relationship to study intervention. All SAEs will be recorded on the appropriate SAE CRF. All SAEs will be followed through resolution or stabilization by a licensed study physician.

9.1.3 Suspected Unexpected Serious Adverse Reactions (SUSAR)
A SUSAR is any SAE where a causal relationship with the study product is at least reasonably possible but is not listed in the Investigator Brochure (IB), and/or Summary of Product Characteristics (SmPC).

9.1.4 Classification of an Adverse Event
The determination of seriousness, severity, and causality will be made by an on-site investigator who is qualified (licensed) to diagnose AE information, provide a medical evaluation of AEs, and classify AEs based upon medical judgment.

9.1.5 Severity of Adverse Events
AEs will be graded according to the following definitions:

- Mild (Grade 1): Events that are usually transient and may require only minimal or no treatment or therapeutic intervention and generally do not interfere with the subject's usual activities of daily living.
- Moderate (Grade 2): Events that are usually alleviated with additional specific therapeutic intervention. The event interferes with usual activities of daily living, causing discomfort but poses no significant or permanent risk of harm to the research subject.
- Severe (Grade 3): Events interrupt usual activities of daily living, or significantly affects clinical status, or may require intensive therapeutic intervention. Severe events are usually incapacitating.
- Life-threatening (Grade 4): Urgent intervention indicated.

AEs characterized as intermittent require documentation of onset and duration of each episode. The start and stop Duration of each reported AE will be recorded on the appropriate CRF. Changes in the severity of an AE will be documented to allow an assessment of the duration of the event at each level of intensity.

9.1.6 Relationship to Study Intervention

For each reported adverse reaction, the SPONSOR must assess the relationship of the event to the study product using the following guideline:

- Related – The AE is known to occur with the study intervention, there is a reasonable possibility that the study intervention caused the AE, or there is a temporal relationship between the study intervention and event. Reasonable possibility means that there is evidence to suggest a causal relationship between the study intervention and the AE.
- Not Related – There is not a reasonable possibility that the administration of the study intervention caused the event, there is no temporal relationship between the study intervention and event onset, or an alternate aetiology has been established.

9.1.7 Time Period and Frequency for Event Assessment and Follow-Up

For this study, all Grade 3 and 4 AEs and all SAEs occurring from the time the informed consent is signed through three months follow-up will be documented, recorded, and reported.

9.1.8 Investigators Reporting of AEs

Information on all grade 3 AEs should be recorded on the appropriate CRF. All clearly related signs, symptoms, and results of diagnostic procedures performed because of an AE should be grouped together and recorded as a single diagnosis. If the AE is a laboratory abnormality that is part of a clinical condition or syndrome, it should be recorded as the syndrome or diagnosis rather than the individual laboratory abnormality. Each AE will also be described in terms of duration (start and stop date), severity, association with the study product, action(s) taken, and outcome.

9.1.9 Serious Adverse Event Reporting

Investigators Reporting of SAEs

Any AE grade 4 that meets a protocol-defined criterion as a SAE must be submitted immediately (within 24 hours of site awareness) in the eCRF. The designated Medical Monitor will review and assess the SAE for regulatory reporting and potential impact on study subject safety and protocol conduct. The Medical Monitor will be available 24/7 on a designated mobile number.

Regulatory Reporting of safety data

All Suspected Unexpected Adverse Reactions (SUSAR) have to be reported, within the legal timeframe, by the sponsor to the National Competent Authority of the Member State concerned. The timelines for expedited initial reporting (day 0) starts as soon as the information containing the minimum reporting criteria has been received by the sponsor. For fatal and life-threatening SUSARs the sponsor should report at least the minimum information as soon as possible and in any case no later than seven days after being made aware of the case. SUSAR which are not fatal and not life-threatening are to be reported within 15 calendar days. If significant new information on an already reported case is received by the sponsor, this information should be reported as a follow-up report within 8 days after being made aware of the relevant complementary information.

In addition, any serious unexpected adverse reaction that is life-threatening (e.g. anaphylaxis, Stevens-Johnson syndrome, aplastic anaemia, or anything comparably strange) must be reported through the WHO study website within 24 hours (SOP 9).

Once a year throughout the clinical trial, the sponsor should submit to the national competent authority and the Ethics Committee of the Member States, an annual safety report.

9.2 Procedures in Case of Emergency
The investigator is responsible for assuring that there are procedures and expertise available to cope with emergencies during the study. The study is not blinded and code breaking procedures is therefore unnecessary.

9.3 Safety Committee
There will be two Data and Safety Monitoring Committees (DSMC), one global and one national. The global is detailed in the core protocol (Appendix 1) while the national DSMC will monitor the safety and evaluate the risk-benefit of the study interventions in the Norwegian patients. The national DSMC will consist of two clinicians and one statistician.

9.4 Protocol deviations
A protocol deviation handling plan will describe reporting procedures for important protocol deviations. Substantial overdosing should be reported within 24 hours in the WHO study website. As the protocol leaves the local doctor fully responsible for all decisions about patient care, including the possibility of discontinuing study medication if this is considered appropriate, the only possible major protocol deviation would be substantial over-dosing with a study drug. If this happens, it should be reported within 24 hours on the study website. The DSMC chair will then decide whether this constitutes a sufficiently major protocol deviation for it to need to be forwarded promptly to the relevant national coordinator and to any relevant ethics committee

10 Statistical methods and data analysis
10.1 Determination of Sample Size
Recruitment will continue until the global core trial is concluded (Appendix 1). Assuming 5% all-cause in-hospital mortality in the standard of care arm and that 2% absolute risk difference is regarded a clinically meaningful difference, 2377 patients in each arm are required to conclude with benefit on a 2.5% two-sided significance level with 90% power. This calculation adjusts for multiple testing using Bonferroni correction. If even more proof is needed, 3412 patients in each arm are needed to conclude with benefit on a 1% two-sided significance level with 95% power. We will not be able to reach these numbers in this trial alone but will rely on the combined analyses of all participating trials in the global consortium.

In this trial we will therefore aim to show a difference in receipt of intensive care. Currently in Norway there are 70 patients in intensive care out of 268 (26%). With a minimum clinically important difference of 10% our goal is to randomise 406 patients in each group to be 90% certain to conclude with benefit on the 2.5% two-sided level.

10.2 Randomization

10.2.1 Allocation- sequence generation
Eligible patients will be allocated in an equal ratio, using a computer randomization procedure. The allocation sequence will be prepared by an independent statistician appointed by the international steering group. The randomisation procedure will accommodate availability of each treatment such that a patient cannot be allocated to an unavailable treatment. There will be no stratification of the allocation sequence, only simple randomisation.

10.2.2 Allocation- procedure to randomize a patient
Eligible patients will be entered into the www.who.int/COVIDcore database including patient baseline details and available treatment arms at the study site. Patients will then be randomised

to one of the available arms. The allocation will be registered in the patient's journal and in the eCRF system (Viedoc). This is an open-label study and no steps to conceal allocation are necessary. The study statistician will be blinded to the randomization allocation for the writing of the national statistical analysis plan (SAP). The authorisations bound to role of the study statistician in the eCRF when reading or downloading data will ensure that the statistician won’t see the treatment allocation until database lock.

10.2.3 Blinding and emergency unblinding
This is an open-label study. However, the staff at the central laboratory at OUS as well as the statistician responsible for analysis of the data will be blinded to the treatment allocation for the writing of the SAP.

10.3 Population for Analysis
The following populations will be considered for the analyses:
- Intention to treat (ITT) population: All randomized participants will be included in the main ITT analyses, regardless of protocol adherence.
- Per-protocol population (PP): Includes all patients in the ITT population having completed the study treatment without major protocol violations and good adherence to the IMP. Criteria for inclusion in the PP population will be specified in the SAP and the final criteria will be defined prior to database lock
- Safety population: Includes all subjects with any safety information after baseline. Patients randomised to Remdesivir or Hydroxychloroquine without receiving any amount of the treatment will be excluded from the safety population.
- Total population: All enrolled participants independent of study arm will be used for additional analyses in the total population.

The primary population is the ITT population.
10.4 Planned analyses
The planned analyses will be detailed in separate statistical analysis plans. There will be two plans, one for the global analyses and one for the analyses of the patients included according to this protocol.

The statistical analyses are planned when the global trial is stopped for either efficacy or futility of the included treatments or when we have reached our recruitment goal of at least 406 patients per arm.

Prior to each statistical analysis, the data in the data base will be exported and the exported data will be locked for further altering of data. A SAP will provide details on the planned statistical analyses. The SAP will be finalized, signed and dated prior to analysis. There will be no efficacy interim analyses according to this protocol.

10.5 Statistical Analysis

10.5.1 Primary analysis
The primary analysis will be identical to the primary analysis according to the WHO core protocol and corresponding statistical analysis plan, restricted to the population included in this add-on trial. The primary analyses assess any effects of treatment allocation on all-cause in-hospital mortality, analyzing separately people who already had severe disease at entry (admitted directly to intensive care) and those who did not.

10.5.2 Secondary analyses

Between group comparisons will be performed for the primary variable on the per-protocol population in addition to secondary efficacy endpoints on both efficacy populations (ITT and PP populations).

The between-group comparisons for secondary variables will be tested as for the primary variable where applicable and additional analyses will be performed based on the following methods (but not limited to):

- Continuous secondary variables will be subject to repeated measures mixed models or appropriate non-parametric alternatives
- Binary response variables will be analysed using logistic regression (possibly adjusting for within-subject dependencies by generalized estimating equations or mixed models) or chi-square/Mantel-Haenszel tests
- Time-to-event variables will be analysed using the Kaplan-Meier method and comparisons between the two groups will be performed using the log rank test, Cox regression analyses or appropriate parametric models such as the gamma or Weibull model.

Unless otherwise specified, all statistical hypotheses will be tested as the primary variable, i.e. with an assessment of superiority of the estimated difference between the groups. All efficacy analyses will be presented with the results from the hypothesis testing (by p-value) in addition to estimates and 95% confidence limits of the treatment effect.

10.5.3 Safety analyses

Safety endpoints include death through three months, SAEs, discontinuation of study infusions, and severe AEs. These events will be analysed univariately and as a composite endpoint. Time-to-event methods will be used for death and the composite endpoint. Each AE will be counted once for a given participant and graded by severity and relationship to COVID-19 or study intervention. AEs will be coded using the current version of the Medical Dictionary for Regulatory Activities (MedDRA). AEs will be presented by system organ class, duration (in days), start- and stop-date. Adverse events leading to premature discontinuation from the study intervention and serious treatment-emergent AEs should be presented either in a table or a listing.

10.5.4 Descriptive statistics

Descriptive statistics will be presented with number and percentages for categorical variables, and means, standard deviation, and range for continuous variables. In case of clearly skewed continuous variables, they will be presented with median, interquartile range (25th and 75th percentiles) and range. Demographics and baseline characteristics will be presented with descriptive statistics without any hypothesis testing.

10.5.5 Missing data

If missing data is regarded as having a significant effect on the conclusions of the trial, sensitivity analyses with different methods for handling missing data will be included. Such methods may include complete case analyses, last observation carried forward, worst case/best case imputation and multiple imputation techniques.

11 Ethical considerations

This study will be conducted in compliance with the principles set out in the Declaration of Helsinki. Where applicable, the principles of Good Clinical Practice (ICH 1996) and other applicable regulations and guidelines will be used to guide procedures and considerations. This protocol will be reviewed and approved by the regional ethical committee and by The Norwegian Medicines Agency (using the EudraCT form) before patients are enrolled in the study.

After approval the study will immediately be registered in ClinicalTrials.gov.

This study is conducted during a disease outbreak. This is a challenging research situation because this falls in the area between clinical care, public health and clinical research (WHO Ethical Review in Disease Outbreak Expert Meeting 2009). Normally research activities are defined by anything conducted outside standard clinical care. The patients included in this study will be treated according to normal practice at the different hospitals, which follow international guidelines in relation to sepsis and ARDS (10, 11).

11.1 Informed Consent

Recruitment of critically ill patients who are not able to consent is a ubiquitous problem in acute and critical care research and there is a clear legal framework under which these patients may be recruited to research studies. In all cases, efforts will be made to obtain informed consent from patients early in the course of illness, before critical illness interferes with their capacity to make decisions and to confirm consent at the earliest point in recovery. Consent forms will be provided in Norwegian or in English if required. If the patient consents in participating in this add-on study he or she automatically consents in participating in the WHO global study.

In this peculiar situation with COVID 19 infected patients, the signed form may itself be a source of infection. To overcome this, a third person (health worker, nurse or physician) will be present while the patient is informed, and independently evaluate and confirm whether the patient is consent competent and voluntary says yes/no to join the study. This will thereafter be documented in the patient's medical record. Thus, a written consent is not required as long as we ensure and document that the patient has been thoroughly informed and voluntary consents to participate.

Due to the patient condition, it is likely that he/she is not capable to sign an informed consent form. Thus, when the patient lacks the capacity to consent to participation, an appropriate representative will be approached. A pdf-file of the consent form will be sent to the patient's representative for reading, who will thereafter be contacted by phone. A third person (health worker, nurse or physician) will join this conversation, and independently evaluate and confirm whether the representative on behalf of the patient, provides an informed consent and voluntary says yes/no to join the study. This will thereafter be documented in the patient's medical record. Staff trained in consent procedures that protect the rights of the patient, and adhere to the ethical principles within the Declaration of Helsinki will be used. Staff will explain the details of the study to the participant or representative and allow them time to discuss and ask questions. The staff will review the informed consent form with the person giving consent and endeavor to ensure understanding of the contents, including study procedures, risks, benefits, the right to withdraw and alternatives to participation. Patient autonomy to withdraw from the study at any time will be respected.

We are sensitive to the fact that some patients or their representatives may feel under an unusually strong moral obligation to participate given the nature of this research and the wide, and often inaccurate, publicity surrounding emerging infections. In view of this, we have tried to make both the potential benefits and limitations of this explorative investigational study clear in the information sheet. In the informed consent form we also stress that participation is entirely voluntary and there is no penalty of any kind for declining to join the study. Balance between public health and research. Patients with emerging infections are commonly the subject of public health investigations. The work proposed here is research and will be clearly presented as such. There is no primary gain to the patient from participating. In designing and describing this

research we are clear that, in accordance with the guiding principles of Good Clinical Practice, the needs and autonomy of the individual are paramount and the potential benefits to wider society do not take precedence.

11.2 Confidentiality

Clinical staff will conduct this study and those involved in the study will ensure that each study participant's privacy and confidentiality is maintained. Participants will not be identified in any published reports of this study. All records will be kept confidential to the extent provided by international and local law. All laboratory specimens, evaluation forms, reports, study protocol, documentation, data and all other information generated will be held in strict confidence. No information concerning the study or the data will be released to any unauthorized third party. Paper and electronic medical records may be accessed during the study to confirm, verify or complete clinical information provided in the case report form. Site files will at all times be accessible only to clinical and research staff. Consent will be sought for investigators to access patient data.

12 Scientific and peer review

The results of the study will be published in a peer-reviewed internationally recognized journal. We are aware that there may be too few patients enrolled in the study, thus our national study may not be able to answer the objectives of the protocol. However, the principal investigators will keep close collaboration with WHO (WHO adaptive master protocol on Clinical Trial of the Safety and Efficacy of Investigational Therapeutics for the Treatment of COVID-19 in Hospitalized Patients) making it likely that the results can be incorporated in a multinational study. This will secure that the data from this study will be
used in a bigger setting and contribute to increased knowledge.

13 References

1. WHO. Global Influenza Strategy 2019-2030 2019.
2. Gordon CJ, Tchesnokov EP, Feng JY, Porter DP, Gotte M. The antiviral compound remdesivir potently inhibits RNA-dependent RNA polymerase from Middle East respiratory syndrome coronavirus. J Biol Chem. 2020 Feb 24.
3. Wang M, Cao R, Zhang L, Yang X, Liu J, Xu M, et al. Remdesivir and chloroquine effectively inhibit the recently emerged novel coronavirus (2019-nCoV) in vitro. Cell research. 2020 Feb 4.
4. Lai CC, Shih TP, Ko WC, Tang HJ, Hsueh PR. Severe acute respiratory syndrome coronavirus 2 (SARS-CoV-2) and coronavirus disease-2019 (COVID-19): The epidemic and the challenges. Int J Antimicrob Agents. 2020 Feb 17:105924.
5. Cortegiani A, Ingoglia G, Ippolito M, Giarratano A, Einav S. A systematic review on the efficacy and safety of chloroquine for the treatment of COVID-19. Journal of critical care. 2020 Mar 10.
6. Bassetti M, Castaldo N, Carnelutti A. Neuraminidase inhibitors as a strategy for influenza treatment: pros, cons and future perspectives. Expert Opin Pharmacother. 2019 Oct;20(14):1711-8.
7. WHO. Master ProtocolA Multi-centre, Adaptive, Randomized, Double-Blind, PlaceboControlled Clinical Trial of the Safety and Efficacy of Investigational Therapeutics for the Treatment of COVID-19 in Hospitalized Patients. 2020.
8. Mulangu S, Dodd LE, Davey RT, Jr., Tshiani Mbaya O, Proschan M, Mukadi D, et al. A Randomized, Controlled Trial of Ebola Virus Disease Therapeutics. The New England journal of medicine. 2019 Dec 12;381(24):2293-303.
9. Sanofi-Aventis. Hydroxychloroquine SmPC. 2020 [cited; Available from: https://www.legemiddelsok.no/_layouts/15/Preparatomtaler/Spc/0000-04266.pdf
10. Rhodes A, Evans LE, Alhazzani W, Levy MM, Antonelli M, Ferrer R, et al. Surviving Sepsis Campaign: International Guidelines for Management of Sepsis and Septic Shock: 2016. Crit Care Med. 2017 Jan 17.
11. Claesson J, Freundlich M, Gunnarsson I, Laake JH, Moller MH, Vandvik PO, et al. Scandinavian clinical practice guideline on fluid and drug therapy in adults with acute respiratory distress syndrome. Acta Anaesthesiol Scand. 2016 Jul;60(6):697-709.

Appendix

A1 The WHO COVID-19 core protocol, version 10

This is attached as a separate document

A2 WHO Standard Operating Procedures and appendix, version 10

This attached as a separate document

A3 National early warning score

National Early Warning Score (NEWS)

PHYSIOLOGICAL PARAMETERS	3	2	1	0	1	2	3
Respiration Rate	≤8		9 - 11	12 - 20		21 - 24	≥25
Oxygen Saturations	≤91	92 - 93	94 - 95	≥96			
Any Supplemental Oxygen		Yes		No			
Temperature	≤35.0		35.1 - 36.0	36.1 - 38.0	38.1 - 39.0	≥39.1	
Systolic BP	≤90	91 - 100	101 - 110	111 - 219			≥220
Heart Rate	≤40		41 - 50	51 - 90	91 - 110	111 - 130	≥131
Consciousness Level				A			V, P, or U

A4 Sofa Score

Table 1. Sequential [Sepsis-Related] Organ Failure Assessment Score[a]

System	Score 0	1	2	3	4
Respiration					
Pao_2/Fio_2, mm Hg (kPa)	≥400 (53.3)	<400 (53.3)	<300 (40)	<200 (26.7) with respiratory support	<100 (13.3) with respiratory support
Coagulation					
Platelets, $\times 10^3/\mu L$	≥150	<150	<100	<50	<20
Liver					
Bilirubin, mg/dL (μmol/L)	<1.2 (20)	1.2-1.9 (20-32)	2.0-5.9 (33-101)	6.0-11.9 (102-204)	>12.0 (204)
Cardiovascular	MAP ≥70 mm Hg	MAP <70 mm Hg	Dopamine <5 or dobutamine (any dose)[b]	Dopamine 5.1-15 or epinephrine ≤0.1 or norepinephrine ≤0.1[b]	Dopamine >15 or epinephrine >0.1 or norepinephrine >0.1[b]
Central nervous system					
Glasgow Coma Scale score[c]	15	13-14	10-12	6-9	<6
Renal					
Creatinine, mg/dL (μmol/L)	<1.2 (110)	1.2-1.9 (110-170)	2.0-3.4 (171-299)	3.5-4.9 (300-440)	>5.0 (440)
Urine output, mL/d				<500	<200

Abbreviations: Fio_2, fraction of inspired oxygen; MAP, mean arterial pressure; Pao_2, partial pressure of oxygen.

[a] Adapted from Vincent et al.[27]

[b] Catecholamine doses are given as μg/kg/min for at least 1 hour.

[c] Glasgow Coma Scale scores range from 3-15; higher score indicates better neurological function.

A4 Clinical Frailty Scale

Clinical Frailty Scale*

1 **Very Fit** – People who are robust, active, energetic and motivated. These people commonly exercise regularly. They are among the fittest for their age.

2 **Well** – People who have **no active disease symptoms** but are less fit than category 1. Often, they exercise or are very **active occasionally**, e.g. seasonally.

3 **Managing Well** – People whose **medical problems are well controlled,** but are **not regularly active** beyond routine walking.

4 **Vulnerable** – While **not dependent** on others for daily help, often **symptoms limit activities.** A common complaint is being "slowed up", and/or being tired during the day.

5 **Mildly Frail** – These people often have **more evident slowing,** and need help in **high order IADLs** (finances, transportation, heavy housework, medications). Typically, mild frailty progressively impairs shopping and walking outside alone, meal preparation and housework.

6 **Moderately Frail** – People need help with **all outside activities** and with **keeping house**. Inside, they often have problems with stairs and need **help with bathing** and might need minimal assistance (cuing, standby) with dressing.

7 **Severely Frail** – **Completely dependent for personal care**, from whatever cause (physical or cognitive). Even so, they seem stable and not at high risk of dying (within ~ 6 months).

8 **Very Severely Frail** – Completely dependent, approaching the end of life. Typically, they could not recover even from a minor illness.

9. **Terminally Ill** - Approaching the end of life. This category applies to people with **a life expectancy <6 months**, who are **not otherwise evidently frail**.

Scoring frailty in people with dementia

The degree of frailty corresponds to the degree of dementia. Common **symptoms in mild dementia** include forgetting the details of a recent event, though still remembering the event itself, repeating the same question/story and social withdrawal.

In **moderate dementia**, recent memory is very impaired, even though they seemingly can remember their past life events well. They can do personal care with prompting.

In **severe dementia**, they cannot do personal care without help.

* 1. Canadian Study on Health & Aging, Revised 2008.
2. K. Rockwood et al. A global clinical measure of fitness and frailty in elderly people. CMAJ 2005;173:489-495.

© 2007-2009. Version 1.2. All rights reserved. Geriatric Medicine Research, Dalhousie University, Halifax, Canada. Permission granted to copy for research and educational purposes only.

DALHOUSIE UNIVERSITY *Inspiring Minds*

A5 Preparation of plasma and serum (study samples)

EDTA-plasma (8mL blood): Collect EDTA-blood into two tubes. Immediately after sampling, mix blood by inverting the tube three times. Place the tube on crushed ice (optional) and centrifuge as soon as possible (within 30 minutes) at 3000*g* for 15 minutes. Isolate plasma (leave the 0.5 cm closest to the cell layer) into three NUNC-tubes (1.8 mL). Freeze immediately -70°C/or -20°C.

Serum (8mL blood): Collect the blood in one red topped tube. Leave it undisturbed at the bench, approximately 30 minutes until coagulation. Then centrifuge at 2000*g* for 10 minutes. Isolate serum (leave the 0.5 cm closest to the cell layer) into three NUNC-tubes (1.8 mL). Freeze immediately -70°C/or -20°C.

Labeling: Every nunc tubes have to be named according to study site number, patient number and sample number.

A6 Participating hospitals and contact information

Hospital	Contact person	E-mail
	**Helse Vest**	
Haukeland	Bjorn Blomberg	bjorn.blomberg@uib.no
Haukeland	Kvåle, Reidar	reidar.kvale@helse-bergen.no
Haraldsplass	Bård Reiakvam Kittang	Bard.Kittang@uib.no
Stavanger	Åse Berg	ase.berg@sus.no
	**Helse Midt**	
St Olavs Hospital, Universitetssykehuset i Trondhjem	Pål Klepstad	Pal.Klepstad@stolav.no
	Raisa Hannula	Raisa.Hannula@stolav.no
Levanger	Skei, Nina Vibeche	NinaVibeche.Skei@helse-nordtrondelag.no
Molde	Tholin, Birgitte	Birgitte.Tholin@helse-mr.no
Ålesund	Dag Arne Lihaug Hoff	Dag.Arne.Lihaug.Hoff@helse-mr.no
	**Helse Nord**	
UNN Tromsø	Anders Benjamin Kildal	Anders.Benjamin.Kildal@unn.no
UNN Tromsø	Vegard Skogen,	Vegard.Skogen@unn.no
Nordlanssykehuset	Hanne Winge Kvarenes	Hanne.Winge.Kvarenes@nordlandssykehuset.no
	**Helse Sør-Øst**	
Ahus	Olav Dalgard	olav.dalgard@medisin.uio.no
Sykehuset i Vestfold	Asgeir Johannessen	UXASOH@siv.no
Telemark Hospital, Skien	Hilde Kristin Skuedal	hskudal@sthf.no
Rikshospitalet	Pål Aukrust	paukrust@ous-hf.no
Rikshospitalet	Andreas Barratt-Due	abarratt@ous-hf.no
Rikshospitalet	Trine Kåsine	TRIKAA@ous-hf.no
Rikshospitalet	Katerina Nezvalova-Henriksen	Katerina.Nezvalova.Henriksen@sykehusapotekene.no
Ullevål	Anne Margarita Dyrhol Riise	a.m.d.riise@medisin.uio.no
Ullevål	Aleksander Rygh Holten	b24675@ous-hf.no
Drammen	Lars Heggelund	Lars.Heggelund@vestreviken.no
Bærum	Anders Aune Tveita	anders.tveita@medisin.uio.no
Kongsberg	Gernot Ernst	bserng@vestreviken.no
Ringerike	Lars Thoresen	lars.thoresen@vestreviken.no
Kristiansand	Mette Haugli	mette.haugli@sshf.no
Arendal	Roy Bjørkholt olsen	Roy.Bjorkholt.Olsen@sshf.no
Kalnes	Prof. Waleed Ghanima	Waleed.Ghanima@so-hf.no
Diakonhjemmet	Leif Erik Vinge	l.e.vinge@medisin.no
Louisenberg	Hedda Hoel	hedda_hoel@hotmail.com
Innlandet Trust	Ragnhild Eiken	Ragnhild.Eiken@sykehuset-innlandet.no
**Microbiology**	Susanne M. R. G. Dudmann	susannmg@medisin.uio.no
	Fredrik Müller	fmuller@ous-hf.no
	Andreas Lind	UXLNDR@ous-hf.no>
**Immunology**	Thor Ueland	thor.ueland@medisin.uio.no
	Tom Eirik Mollnes	t.e.mollnes@medisin.uio.no
	Bente Halvorsen	b.e.halvorsen@medisin.uio.no

A7 Flow chart

The NOR Solidarity multicenter trial:

FLYTSKJEMA

				EKG: Inklusjon Dag 1	Daglig	HUSK EKG[8]: Dag 3-5	HUSK EKG[8]: Dag 7-9	Innlagt > 10 dager	Innlagt ICU	Dato for utskrivelse /DØD[3]	AE Grad 3[2]	SUSARS[1]	Død 28 dager	Status 3 mnd[4]
WHO COVID 19	Inklusjon	Påvist COVID-19		X										
		> 18 år*		X										
		Informert samtykke		X										
	Randomisering	Gå inn på www.who.int/COVIDcore : Pas identitet, komorbiditet, kliniske status		X										
		Det blir utlevert et pasientnummer og pas. randomisert til en behandlingsgruppe[5]		X										
	Outcome/hendelser	Dato for utskrivelse, evt. død, evt. intensiv/MV							Ja/Nei	X		X		
WHO NOR-COVID 19	Kliniske data	**eCRF (Sengepost og intensiv registreres daglig)[NB!]**		X	X				Ja/Nei	X	X		X	X
		**SOFA score**		X		X	X							
		**Frailty Score**		X										
	Lab og billed diagnostikk	**Medisinsk biokjemi, sengepost**	Standard pakke jfr. CRF[6]	X		X	X	Hver 3.dag						
		**Medisinsk biokjemi, Intensiv**			X									
		**Blodgass, Intensiv**			X				X					
		**Virus clearance SARS-CoV-2**	Nasopharynx prøve	X		X	X	Hver 3.dag						
		**Mikrobiologi**	Anfør i eCRF aktuelle co-infeksjoner og hvor positiv prøve er tatt fra											
		**Billeddiagnostikk**	Ekko, Rtg. og CT funn: Angi funn forenlig med mest alvorlige kliniske situasjon under oppholdet											
		**3 måneders kontroll:**	QOL ED50											
	Biobanking	**Blod**	**Prøvetakingsrør / Antall / Volum**											
			EDTA plasma / 3 / rør á 4 ml	X		X	X	X1/uke		X				
			Serum / 1 / rør á 8 ml	X		X	X	X1/uke		X				
		**Dyp luftveisprøve**	Virus clearance SARS-CoV-2, hver tredje dag dersom intubert											

*) Kvinner i gravid alder må utføre graviditetstest, positiv prøve ekskluderer fra deltagelse

[1]) SUSARS og alvorlige avvik skal meldes WHOwww.who.int/COVIDcore innen 24 time

[2]) Adverse Events grad 3 skal nøye noteres i CRF

[3]) Dato for utskrivelse av sykehus/Intensiv eller død sykehus/intensiv må anføres

[4]) Telefonisk oppfølging. Klinisk konsulatsjon enkelte sentre som bestemmes senere

[5]) Behandling gis 10 dager; Hydroxychloroquine, Remdesivir eller intet (kan bli flere studiearmer)

[6]) Hb, trc, CRP, hvite m. Diff, procalcitonin, haptoglobin, APTT, Fibrinogen, D-dimer, INR, Na, K, Kreatinin, Urea, Ferritin, LD, bilirubin, ASAT, ALAT, ALP, Albumin, Amylase, CK-MB, troponin, pro-BNP, CK + immunglobuliner v/innleggelse **NB! 1)** Forståelse for at ikke alle kan ta alt, men prøv å sikre så komplette datasett som mulig. 2) Sengepost kan og registrere MBK hyppigere enn angitt.

[8]) Spesielt pasienter som mottar hyfroxyklorokin må følges med EKG

NB!

Sengepost: Daglig registrering av SpO_2, Oksygenbehandling Ja/nei, antall liter O_2

Intensiv: Daglig registrering av respirasjonstøtte, blodgass, sirkulasjonstøtte og annen organsupport
